# Supplementary material for: Effect of specificity of health expenditure questions in the measurement of out-of-pocket health expenditure: evidence from field experimental study in Ghana
Source: BMJ Open. 2021 May 3;11(5):e042562. doi: 10.1136/bmjopen-2020-042562 (PMC8098927; doi:10.1136/bmjopen-2020-042562)
Supplement: Supplementary data [file bmjopen-2020-042562supp001.pdf]

Supplementary material 1

Data collection instrument design and matching procedure

Ghana Living Standards Survey 6 (GLSS6)

The Ghana Living Standards survey (GLSS) is a multi-purpose household survey instituted by the World Bank to collects information on many different dimensions of living conditions, including education, health, employment and household expenditure on food and non-food items. The GLSS 6 is the sixth edition of the survey conducted in 2012/2013 (GLSS6 Report, 2014). The survey instrument has one large module on household consumption reported by a single respondent and structured around food versus non-food expenditures (health expenditure included) using similar recall periods for all food and non-food divisions regardless of their relevance in gathering information on health. The consumption module in the survey is comprehensive in covering household consumption items. The structure of the consumption module follows the Classification of individual Consumption According to Purpose (COICOP 1999) classifications. COICOP-1999 is the international reference classification of household expenditure which aims at providing a framework of homogeneous categories of goods and services considered a function or purpose of household consumption expenditure. Following this classification, the GLSS6 has 251 items in the consumption module, of these; about 7% (17 items) are questions asking about health expenditure. The GLSS6 adopted the second level of disaggregation of COICOP-1999 for collecting information on Health expenditure in the consumption module but COICOP-1999 was of little guidance when there is an interest in refining the information on health expenditure beyond the second level of disaggregation. Previous analysis (Lavado, Brooks & Hanlon, 2013) has shown that as the total number of health and non-health consumption expenditure question increases, the average health expenditure budget share tends to either decrease or increase and as such given the structure of the GLSS, it is difficult to assess the accuracy of the information collected. The GLSS has been the primary source of data for estimating OOPs in Ghana.

Specificity of the iHOPE household survey instrument

The starting point to develop questions on OOPs was the GLSS6 but with the objective to align it with division 6 of COICOP-2018 which was refined to better cover all the different type of health products and services available to the population. Version 1 of the OOPs questionnaire captured six major categories namely medicines, health products, preventive care services, other outpatient care services, inpatient care services and other health services to obtain a total of 11 health items. The second questionnaire further expanded all main six categories (Table 1) to a total of 44 health items. In the third questionnaire version only questions on OOPs for medicines and health products were expanded to arrive at a total of 56 health items for version three. Main difference between questions in OOPs included in the GLSS6 and those developed for this study are listed in Table 1.

The new developed questions on out-of-pocket health care expenditure were then included at the end of GLSS6 consumption module. Questions related to the provider were asked to each person reporting an OOP in order to be able to compare provider and household information. The final instruments included in addition to the consumption expenditure module a household roster, a module on housing characteristics and household assets. Figure 1 shows the generic structure of the developed instrument.

Figure 1: Structure of survey instruments

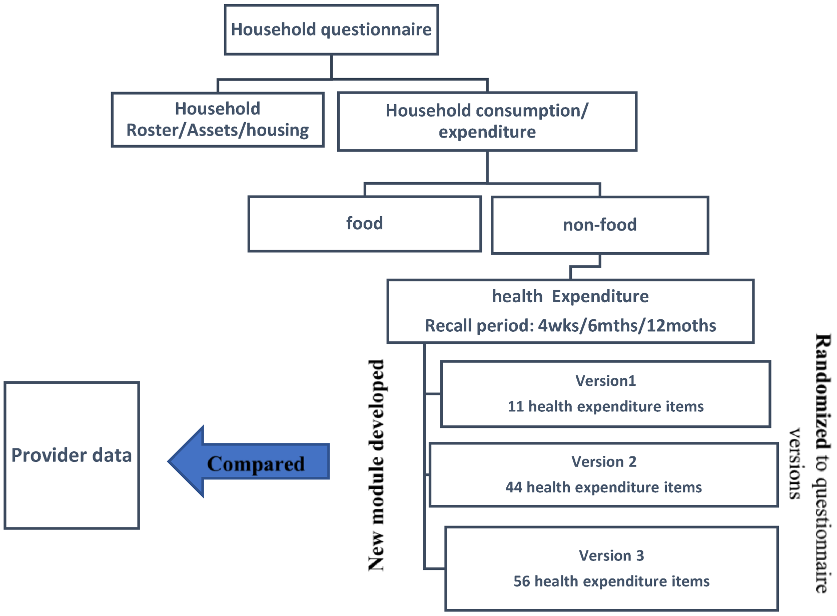

Table 1: Specificity of the health expenditure list

Table 1GLSS 6	Version 1	Version 2	Version 3
Based on COICOP-1999, cover all major classes of health products and services.	covers all major classes of health products and services are redefined in COICOP-2018		
Total number of questions on OOPs for health is 24 split as follows:	Total number of questions on OOPs for health is 11 split as follows:	Total number of questions on OOPs for health is 44. Expand version 1 as follows:	Total number of questions on OOPs for health is 56. Expand version 2 as follows:
Pharmaceutic products and other medical products – 6 questions	Medicines – 2 questions to distinguish herbal from non-herbal medicine	Expands questions on non-herbal medicines (7 questions)	Questions on non-herbal medicines are split into prescribed and non-prescribed medicines
MEDICAL PRODUCTS, APPLIANCES AND EQUIPMENT – 2 questions to differentiate appliance and equipment from assistive products	Health products – 2 questions to distinguish assistive products from an overall category on medical diagnostic products, prevention and protective devices.	Differentiates between purchase of assistive products and repair/rental/maintenance of assistive products	Within purchase and repair of assistive products a distinction is made between those for vision and hearing versus mobility and daily living. The overall category on medical diagnostic products, prevention and protective devices is split into 3.
Outpatient medical services – 14 questions to differential type of health care provider and public/private	Outpatient medical services – 4 questions to differentiate within preventive services immunization/vaccination services from other preventive services (e.g. check-ups); dental outpatient services from other outpatient services.	Immunization vaccination services differentiate between those related to maternal and child care from other vaccination services.<br><br>Other preventive services are split into 3 categories<br>Dental care services and other outpatient services are split by type of facility (hospital versus outpatient setting) and OOPs for consultation are differentiated from OOPs for laboratory tests, imagining services when possible. In addition for other outpatient services the type of health care provider is also distinguished (e.g. specialist, general doctor)	
Hospital services – 2 questions to distinguish public/private	Inpatient services – 2 questions to differentiate long term inpatient care from other overnight stays	Details on inpatient long term care and other overnight stays are gathered by the means of	
	Other health services - 1 question to capture emergency transportation and emergency rescue services	Emergency transportation is split into 2 categories 7 questions covering fees, products, tests and emergency transportation related to the inpatient treatment	

Provider data collections

Within the Ghana health care system, public health providers who are managed by the government keep patient records as part of routine activities while most private providers either kept minimal transactional records or no records at all. To generate accurate data for comparison, we developed a template (Supplementary material 2) and trained private provider owners on how to use it to collect patient information. The main fields in the template included name, address, phone number, referral status, reason for consultation and cost of treatment/service. For public providers where patient data was already

recorded, trained field workers completed the template by review and extracting relevant information from the provider records. We therefore purposely selected major health care providers within the demographic surveillance site to obtain the provider data. The criterion for selecting the providers was based on the availability of transactional data or a care-taker who could record details of the transactions from clients in the case of private providers. The public health providers selected include; one hospital, one clinic and seven public health centres. For the private health care providers, ten high volume pharmacy and license chemical shops met our selection criteria and were selected.

Matching

Matched samples in this study refer to households that were linked to their provider records. For any households that reported positive expenditure on any of the health expenditure items, corresponding health provider data was obtained from the provider records using details about the provider obtained from the respondents. The linked household-provider data formed a matched sample used in our analysis. The matching of household and provider data was done at the individual household member level and by spending category. Due to challenges of completeness and accuracy of provider details provided by respondents and possible errors in recording patient details at the provider, some households could not be matched to provider data and were therefore excluded from the analysis. The challenges that influenced the completeness and accuracy of provider data are discussed in another paper "Challenges in linking household health expenditure records to provider records" (Agorinya et al, in prep)

Table 2: OOPs that successfully matched with health provider records by type of health provider

Type of provider	Total number of clients attending provider	proportion of clients with linked records to household
Hospital	453	46.8
Community Health Centre	195	55.4
CHPS	196	90.3
Clinic	58	27.6
Chemical Shop	194	71.1
Diagnostic laboratory	29	82.8
Hawker/Vendor/ Mobile Van	25	0.0
General local shop	81	33.3
Other	16	12.5
Pharmacy	155	73.6
Total	1402	59

Table 3: OOPs that successfully matched with health provider records by type of care received

Spending category	Total number of cases	Number of cases matched	Proportion of cases matched
Inpatient care	339	159	47.2
Out-patient	551	351	63.4
Medicines	468	286	61.6
Preventive care	32	22	68.8
Medical products	7	1	14.3
Total	1397	820	59
