# Supplementary material for: Effect of specificity of health expenditure questions in the measurement of out-of-pocket health expenditure: evidence from field experimental study in Ghana
Source: BMJ Open. 2021 May 3;11(5):e042562. doi: 10.1136/bmjopen-2020-042562 (PMC8098927; doi:10.1136/bmjopen-2020-042562)
Supplement: Supplementary data [file bmjopen-2020-042562supp002.pdf]

Supplementary material 2

COICOP 2018 based Levels of disaggregation of Health items

Type of information	*Version-1*<br>Level of Disaggregation – 11 Health items	Recall periods: D- days/ M-Months				
		15d	30d	3M	6M	12M
	COICOP code 06.2.3 Inpatient care services					
To be asked in bold<br>Examples are given in brackets for more see explanatory notes.<br>**If helpful can add at the end of the explanations: for patients with disabilities, the elderly (or those who requires permanent surveillance or constant help due to limited functional capacity)**	**I.LONG. Medical treatment and / or care that required overnight stay in a nursing home; (medical convalescent homes; palliative care establishments) or any other long term care medical facility**				X	X
	**I.CURR. Medical and dental treatment that required an overnight stay in any type of facility** (e.g. hospitals, clinics) **excluding long term care medical facility**				X	X
Inclusion/Exclusion criteria to be specified when asking about the amount<br>Applies to I.LONG & I.CURR	Includes payments for all medical services, diagnostic and laboratory tests, medicines and medical products needed during the overnight stay. Also include emergency transportation services and emergency rescue.<br>Excludes: non-emergency transportation and non-medical costs for patient's relative.					
	COICOP code 06.2.1 Preventive care services					
	**P.IMMV. Immunization/vaccination services including for maternal and child care**			X	X	
Inclusion/Exclusion criteria to be specified when asking about the amount	Includes ; travel and tourism vaccination as well as any other immunization/vaccination service.					
	**P.OTHR. Other preventive services such as prenatal/postnatal care, child growth and development visits, family planning, screening, tests, consultations to detect communicable or non-communicable diseases before symptoms appear** (e.g. diabetes, heart problems)			X	X	
Inclusion/Exclusion criteria to be specified when asking about the amount<br>Applies to both P.IMMV & P.OTHR	Includes diagnostic and laboratory tests needed to provide preventive services but exclude payments for the vaccine itself when separately invoiced from the service.					
	COICOP code 06.4.2 Emergency transportation and emergency rescue					
Alternative wording: transportation for medical emergency reasons (e.g. by ambulance)	**O.EMER. Patient emergency transportation services and emergency rescue services (excluding those associated with an overnight stay)**	X	X			
Inclusion/Exclusion criteria to be specified when asking about the amount	Excludes non-emergency transportation services					
	COICOP code 06.2.2 & 06.2.3 Outpatient dental & other outpatient services					
	**O.DENT. Dental consultations and services that did not require an overnight stay;**	X	X			
Inclusion/Exclusion criteria to be specified when asking about the amount	Includes dental diagnostics services and laboratory tests needed to provide outpatient dental services (e.g. X-rays, blood tests)<br>For any dental illness, disease, injury or health problem; from any type of provider; inside or outside a hospital setting					
	**O.CRRL. other medical consultations and services than dental and preventive that did not require an overnight stay**	X	X			
Inclusion/Exclusion criteria to be specified when asking about the amount	Includes any diagnostic and laboratory test needed to provide outpatient medical services (e.g. X-rays, blood/urine tests), but excludes emergency transportation services and emergency rescue					
	COICOP code 06.1.1					
	**M.HERH. Herbal medicines (tablets or syrups) and homeopathic products for consumption outside a health facility or institution.**	X	X			
	**M.MVCP. Medicines** (branded, generic), **vaccines, oral contraceptives, vitamins and minerals and other pharmaceutical preparations for consumption outside a health facility or institution.**	X	X			
	COICOP code 06.1. 2 medical diagnostic products, prevention and protective devices					
Inclusion/Exclusion criteria to be specified when asking about the amount	**D.** (Pregnancy tests, incontinence products and absorbent including diapers for the aging population, inhalers, mechanical contraceptives; insecticide treated mosquito nets, blood pressure devices) **and other medical health products for personal use outside a health facility or institution**.	X	X			
Only applies to some diagnostic products	Includes repair, rental and maintenance					
	COICOP code 06.1.3 Assistive products for vision, hearing, mobility and daily living.					

	**A. Purchase, repair, rental/maintenance of** (glasses for vision; hearing aids; crutches & wheelchairs; therapeutic footwear; walkers; pressure relief mattresses) **and all other assistive health products** .				X	X
**Type of information**	***Version-2***<br>**Level of Disaggregation – 44 Health items**	**Recall periods:<br>D- days/ M-Months**				
		**15d**	**30d**	**3M**	**6M**	**12M**
	**COICOP code 06.2.3 Inpatient care services**					
**To be asked in bold**<br>Examples are given in brackets for more see explanatory notes<br>**If helpful can add at the end of the explanations: for patients with disabilities, the elderly (or those who requires permanent surveillance or constant help due to limited functional capacity)**	**I.LONG. Medical treatment and / or care that required overnight stay in a nursing home; (medical convalescent homes; palliative care establishments) or any other long term care medical facility**<br>**If yes**					
	**I.LONG.SP.1 medical services during the overnight long term care**				X	X
	**I.LONG.SP.2 medicines during the overnight long term care**				X	X
	**I.LONG.SP.3 medical products during the overnight long term care**				X	X
	**I.LONG.DT. diagnostic and laboratory tests**				X	X
	**I.LONG.NM. Non-medical cost for the patient (cooking, cleaning, accommodation) during the overnight long term care**				X	X
	**I.LONG.ER.1 Emergency transportation and rescue services by ambulance or other vehicles specially adjusted for medical purposes**				X	X
	**I.LONG.ER.2 Emergency transportation services and rescue by ordinary vehicles or airplanes (not specially adjusted for a medical purpose)**				X	X
	**I.CURR Medical and dental treatment that required an overnight stay in any type of facility** (e.g. hospitals, clinics) **excluding long term care medical facility**<br>**If yes**					
	**I.CURR.SP.1. medical, dental services during overnight stay**				X	X
	**I.CURR.SP.2. medicines for medical or dental treatment during overnight stay**				X	X
	**I.CURR.SP.3. medical products for medical or dental treatment during overnight stay**				X	X
	**I.CURR.DT. diagnostic and laboratory tests for medical or dental treatment during overnight stay (e.g. x-rays, scans, blood tests)**				X	X
	**I.CURR.NM. Non-medical costs for the patient (cooking, cleaning, accommodation)**				X	X
	**I.CURR.ER.1. emergency transportation and rescue services by ambulance or other vehicles specially adjusted for medical purposes**				X	X
	**I.CURR.ER.2. Emergency transportation services and rescue by ordinary vehicles or airplanes (not specially adjusted for a medical purpose)**				X	X
**Inclusion/Exclusion criteria to be specified when asking about the amount**<br>Applies to both I.LONG.SP.1 & I.CURR.SP.1	*If possible* exclude diagnostic and laboratory tests during the overnight stay I.LONG.DT/ I.CURR.DT. and emergency transportation services and emergency rescue (I.LONG.ER / I.CURR.ER)					
Applies to all I.LONG. & I.CURR	Excludes: non-emergency transportation and non-medical costs for patient's relative.					
	**COICOP code 06.2.1 Preventive care services**					
	**P.IMMV.MC. Immunization/vaccination services for maternal and child care**			X	X	
	**P.IMMV.OV. Travel and tourism vaccination, any other compulsory or voluntary immunization/vaccination service.**			X	X	
**Inclusion/Exclusion criteria to be specified when asking about the amount**<br>Applies to P.IMMV.OV	Includes; travel and tourism vaccination as well as any other immunization/vaccination service.					
Applies to both P.IMMV.MC and P.IMMV.OV	Excludes payments for the vaccine itself when separately invoiced from the service.					
	**P.OTHR. Other preventive services than immunization/vaccination**					
	**P.OTHR.GH.1 Family planning, counselling, prenatal/postnatal care services for both the mother and new born (during the six weeks or 42 days)**			X	X	
	**P.OTHR.GH.2 Child growth and development consultation visits and any other consultations to monitor "good" health of children and adults**			X	X	
	**P.OTHR.DI. screening, tests, consultations to detect communicable or non-communicable diseases before symptoms appear** (e.g. diabetes, heart problems)			X	X	
**Inclusion/Exclusion criteria to be specified when asking about the amount**<br>Applies to all P.IMMV & P.OTHR	Includes diagnostic and laboratory tests needed to provide preventive services					
	**COICOP code 06.4.2 Emergency transportation and emergency rescue**					
**Alternative wording: transportation for medical emergency reasons (e.g. by ambulance)**	**O.EMER. Patient emergency transportation services and emergency rescue services (excluding those associated with an overnight stay)**					

	O.EMER.AV. by ambulance or other vehicles specially adjusted for medical purpose	X	X			
	O.EMER.NA. by ordinary vehicles or airplanes (not specially adjusted for a medical purpose)	X	X			
Inclusion/Exclusion criteria to be specified when asking about the amount	Excludes non-emergency transportation services					
	COICOP code 06.2.2 & 06.2.3 Outpatient dental & other outpatient services					
	O.DENT Outpatient dental care					
	In a hospital setting					
	O.DENT.CS.1 Dental consultations and services that did not require an overnight stay in a hospital setting	X	X			
	O.DENT.DT.1. Diagnostic and laboratory tests needed to provide dental consultations and services in a hospital setting	X	X			
	Outpatient settings (e.g. private practice, office, medical center, clinics, polyclinics)					
	O.DENT.CS.2. Dental consultations and services that did not require an overnight stay Outpatient settings (e.g. private practice, office, medical center, clinics, polyclinics)	X	X			
	O.DENT.DT.2. Diagnostic and laboratory tests needed to provide dental consultations and services Outpatient settings (e.g. private practice, office, medical center, clinics, polyclinics)	X	X			
Inclusion/Exclusion criteria to be specified when asking about the amount Applies to O.DENT.CS..1 & O.DENT.CS.2	Excludes dental diagnostics services and laboratory tests needed to provide outpatient dental services (e.g. X-rays, blood tests)					
	O.CRRL. Other medical consultations and services than dental and preventive that did not require an overnight stay					
	In a hospital setting					
	O.CRRL.CS.1.1 consultations and services of specialists (paediatricians, surgeons, cardiologists, ophthalmologist, mental health)	X	X			
	O.CRRL.CS.1.2 consultation and services of general doctors	X	X			
	O.CRRL.CS.1.3 consultation and services of nurses, midwifes and other health care practitioner	X	X			
	O.CRRL.DT.1 diagnostic and laboratory tests needed to provide other medical services that did not require an overnight stay in a hospital setting	X	X			
	Outpatient settings (e.g. private practice, office, medical center, clinics, polyclinics)					
	O.CRRL.CS.2.1 consultations and services of specialists (paediatricians, surgeons, cardiologists, ophthalmologist, mental health)	X	X			
	O.CRRL.CS.2.2 consultation and services of general doctors	X	X			
	O.CRRL.CS.2.3 consultation and services of nurses, midwifes and other health care practitioner	X	X			
	O.CRRL.DT.2 diagnostic and laboratory tests needed to provide other medical services that did not require an overnight stay outside a hospital setting	X	X			
Inclusion/Exclusion criteria to be specified when asking about the amount Applies to all O.CRRL.CS.1 & O.CRRL.CS.2	excludes diagnostic and laboratory test needed to provide outpatient medical services (e.g. X-rays, blood/urine tests)					
	COICOP code 06.1.1					
	M.HERH. Herbal medicines (tablets or syrups) and homeopathic products for consumption outside a health facility or institution.	X	X			
	M.MVCP. Medicines (branded, generic), vaccines, oral contraceptives, vitamins and minerals for consumption outside a health facility or institution.					
	M.MVCP.IA. antibiotics	X	X			
	M.MVCP.IO.Other medicines (branded, generic, homeopathic) to treat (presumed or diagnosed) bacterial infections (e.g. malaria, diarrhoeas, dysentery, increased frequency of stools with or without blood and mucus in stools; worms infestations)	X	X			
	M.MVCP.CD. medicines to treat (presumed or diagnosed) non-communicable diseases or chronic diseases (e.g. diabetes, hypertension)	X	X			
	M.MVCP.FP. oral contraceptives and contraceptives in the form of injections	X	X			
	M.MVCP.SY medicines to treat fevers, pain and other symptoms (e.g. nausea; vomiting, constipation; inflammation)	X	X			
	M.MVCP.VM. vitamins, mineral	X	X			
	M.MVCP.OM. other medicines and pharmaceutical preparations not elsewhere specified	X	X			
	COICOP code 06.1. 2 medical diagnostic products, prevention and protective devices					

	**D.** (Pregnancy tests, incontinence products and absorbent including diapers for the aging population, inhalers, mechanical contraceptives; insecticide treated mosquito nets, blood pressure devices) **and other medical health products for personal use outside a health facility or institution**.	**X**	**X**			
Inclusion/Exclusion criteria **to be specified when asking about the amount** Only applies to some diagnostic products	Includes repair, rental and maintenance					
	**COICOP code 06.1.3 Assistive products for vision, hearing, mobility and daily living.**					
	**A.PURC. Purchase of** (glasses for vision; hearing aids; crutches & wheelchairs; therapeutic footwear; walkers; pressure relief mattresses) **and all other assistive health products** .				**X**	**X**
	**A.RRMN. Repair, rental/maintenance of** (glasses for vision; hearing aids; crutches & wheelchairs; therapeutic footwear; walkers; pressure relief mattresses) **and all other assistive health products** .				**X**	**X**

Type of information	***Version-3*** **Level of Disaggregation – 56 Health items**	Recall periods: D- days/ M-Months				
		**15d**	**30d**	**3M**	**6M**	**12M**
	**COICOP code 06.2.3 Inpatient care services**					
**To be asked in bold**<br>Examples are given in brackets for more see explanatory notes<br>**If helpful can add at the end of the explanations: for patients with disabilities, the elderly (or those who requires permanent surveillance or constant help due to limited functional capacity)**	**I.LONG. Medical treatment and / or care that required overnight stay in a nursing home; (medical convalescent homes; palliative care establishments) or any other long term care medical facility**<br>**If yes**					
	**I.LONG.SP.1 medical services during the overnight long term care**				X	X
	**I.LONG.SP.2 medicines during the overnight long term care**				X	X
	**I.LONG.SP.3 medical products during the overnight long term care**				X	X
	**I.LONG.DT. diagnostic and laboratory tests**				X	X
	**I.LONG.NM. Non-medical cost for the patient (cooking, cleaning, accommodation) during the overnight long term care**				X	X
	**I.LONG.ER.1 Emergency transportation and rescue services by ambulance or other vehicles specially adjusted for medical purposes**				X	X
	**I.LONG.ER.2 Emergency transportation services and rescue by ordinary vehicles or airplanes (not specially adjusted for a medical purpose)**				X	X
	**Medical and dental treatment that required an overnight stay in any type of facility** (e.g. hospitals, clinics) **excluding long term care medical facility**<br>**If yes**					
	**I.CURR.SP.1. medical, dental services during overnight stay**				X	X
	**I.CURR.SP.2 medicines for medical or dental treatment during overnight stay**				X	X
	**I.CURR.SP.3 medical products for medical or dental treatment during overnight stay**				X	X
	**I.CURR.DT. diagnostic and laboratory tests for medical or dental treatment during overnight stay (e.g. x-rays, scans, blood tests)**				X	X
	**I.CURR.NM. Non-medical costs for the patient (cooking, cleaning, accommodation)**				X	X
	**I.CURR.ER.1.emergency transportation and rescue services by ambulance or other vehicles specially adjusted for medical purposes**				X	X
	**I.CURR.ER.2. Emergency transportation services and rescue by ordinary vehicles or airplanes (not specially adjusted for a medical purpose)**				X	X
**Inclusion/Exclusion criteria to be specified when asking about the amount**<br>Applies to both I.LONG.SP.1 & I.CURR.SP.1	*If possible* exclude diagnostic and laboratory tests during the overnight stay I.LONG.DT/ I.CURR.DT. and emergency transportation services and emergency rescue (I.LONG.ER / I.CURR.ER)					
Applies to all I.LONG. & I.CURR	Excludes: non-emergency transportation and non-medical costs for patient's relative.					
	**COICOP code 06.2.1 Preventive care services**					
	**P.IMMV.MC Immunization/vaccination services for maternal and child care**			X	X	
	**P.IMMV.OV Travel and tourism vaccination, any other compulsory or voluntary immunization/vaccination service.**			X	X	
**Inclusion/Exclusion criteria to be specified when asking about the amount**<br>Applies to P.IMMV.OV	Includes; travel and tourism vaccination as well as any other immunization/vaccination service.					
Applies to both P.IMMV.MC and P.IMMV.OV	Excludes payments for the vaccine itself when separately invoiced from the service.					
	**P.OTHR. Other preventive services than immunization/vaccination**					
	**P.OTHR.GH.1 Family planning, counselling, prenatal/postnatal care services for both the mother and new born (during the six weeks or 42 days)**			X	X	
	**P.OTHR.GH.2 Child growth and development consultation visits and any other consultations to monitor "good" health**			X	X	
	**P.OTHR.DI. screening, tests, consultations to detect communicable or non-communicable diseases before symptoms appear** (e.g. diabetes, heart problems)			X	X	
**Inclusion/Exclusion criteria to be specified when asking about the amount**<br>Applies to all P.IMMV & P.OTHR	Includes diagnostic and laboratory tests needed to provide preventive services					
	**COICOP code 06.4.2 Emergency transportation and emergency rescue**					
**Alternative wording: transportation for medical emergency reasons (e.g. by ambulance)**	**O.EMER. Patient emergency transportation services and emergency rescue services (excluding those associated with an overnight stay)**					

	**O.EMER.AV by ambulance or other vehicles specially adjusted for medical purpose**	X	X			
	**O.EMER.NA by ordinary vehicles or airplanes (not specially adjusted for a medical purpose)**	X	X			
**Inclusion/Exclusion criteria to be specified when asking about the amount**	Excludes non-emergency transportation services					
	**COICOP code 06.2.2 & 06.2.3 Outpatient dental & other outpatient services**					
	**O.DENT Outpatient dental care**					
	**O.DENT.CS.1 Dental consultations and services that did not require an overnight stay in a hospital setting**	X	X			
	**O.DENT.DT.1 Diagnostic and laboratory tests needed to provide dental consultations and services in a hospital setting**	X	X			
	**O.DENT.CS.2 Dental consultations and services that did not require an overnight stay Outpatient settings (e.g. private practice, office, medical center, clinics, polyclinics)**	X	X			
	**O.DENT.DT.2 Diagnostic and laboratory tests needed to provide dental consultations and services Outpatient settings (e.g. private practice, office, medical center, clinics, polyclinics)**	X	X			
**Inclusion/Exclusion criteria to be specified when asking about the amount** Applies to O.DENT.CS.1 & O.DENT.CS.2	Excludes dental diagnostics services and laboratory tests needed to provide outpatient dental services (e.g. X-rays, blood tests)					
	**O.CRRL. Other medical consultations and services than dental and preventive that did not require an overnight stay**					
	**In a hospital setting**					
	**O.CRRL.CS.1.1 consultations and services of specialists (paediatricians, surgeons, cardiologists, ophthalmologist, mental health)**	X	X			
	**O.CRRL.CS.1.2 consultation and services of general doctors**	X	X			
	**O.CRRL.CS.1.3 consultation and services of nurses, midwifes and other health care practitioner**	X	X			
	**O.CRRL.DT.1 diagnostic and laboratory tests needed to provide other medical services that did not require an overnight stay in a hospital setting**	X	X			
	**Outpatient settings (e.g. private practice, office, medical center, clinics, polyclinics)**					
	**O.CRRL.CS.2.1 consultations and services of specialists (paediatricians, surgeons, cardiologists, ophthalmologist, mental health)**	X	X			
	**O.CRRL.CS.2.2 consultation and services of general doctors**	X	X			
	**O.CRRL.CS.2.3 consultation and services of nurses, midwifes and other health care practitioner**	X	X			
	**O.CRRL.DT.2. diagnostic and laboratory tests needed to provide other medical services that did not require an overnight stay outside a hospital setting**	X	X			
**Inclusion/Exclusion criteria to be specified when asking about the amount** Applies to all O.CRRL..CS1 & O.CRRL..CS.2	excludes diagnostic and laboratory test needed to provide outpatient medical services (e.g. X-rays, blood/urine tests)					
	**COICOP code 06.1.1**					
	**M.HERH. Herbal medicines (tablets or syrups) and homeopathic products for consumption outside a health facility or institution.**					
	**M.HERH.PR prescribed**	X	X			
	**M.HERH.OC over-the-counter (self-prescription)**	X	X			
	**M.MVCP. Medicines** (branded, generic)**, vaccines, oral contraceptives, vitamins and minerals for consumption outside a health facility or institution.**					
	**prescribed**					
	**M.MVCP.PR.IA. antibiotics**	X	X			
	**M.MVCP.PR.IO. Other medicines (branded, generic, homeopathic) to treat (presumed or diagnosed) bacterial infections (e.g. malaria, diarrhoeas, dysentery, increased frequency of stools with or without blood and mucus in stools; worms infestations)**	X	X			
	**M.MVCP.PR.CD. medicines to treat (presumed or diagnosed) non-communicable diseases or chronic diseases** (e.g. diabetes, hypertension)	X	X			
	**M.MVCP.PR.FP. oral contraceptives and contraceptives in the form of injections**	X	X			
	**M.MVCP.PR.SY. medicines to treat fevers, pain and other symptoms (e.g. nausea; vomiting, constipation; inflammation)**	X	X			
	**M.MVCP.PR.VM. vitamins, mineral**	X	X			
	**M.MVCP.PR.OM. other prescribed medicines and pharmaceutical preparations not elsewhere specified**	X	X			

	**over-the-counter (self-prescription)**					
	**M.MVCP.OC.IA. antibiotics**	X	X			
	**M.MVCP.OC.IO Other medicines (branded, generic, homeopathic) to treat bacterial infections (e.g. malaria, diarrhoeas, dysentery, increased frequency of stools with or without blood and mucus in stools; worms infestations)**	X	X			
Examples on NCD should list the most prevalent in country/site	**M.MVCP.OC.CD medicines to treat (presumed or diagnosed) non-communicable diseases or chronic diseases** (e.g. diabetes, hypertension)	X	X			
	**M.MVCP.OC.FP oral contraceptives and contraceptives in the form of injections**	X	X			
	**M.MVCP.OC.SY medicines to treat fevers, pain and other symptoms (e.g. nausea; vomiting, constipation; inflammation)**	X	X			
	**M.MVCP.OC.VM vitamins, mineral**	X	X			
	**M.MVCP.OC.OM other self-prescribed medicines and pharmaceutical preparations not elsewhere specified**	X	X			
	**COICOP code 06.1. 2 medical diagnostic products, prevention and protective devices for personal use outside a health facility or institution**					
	**D.DIAG.** (pregnancy tests; thermometers, glucose-meters, blood pressure meters) **and other medical diagnostic products**	X	X			
	**D. PREP** condoms and other mechanical contraceptive devices, masks , medicinal stockings (e.g. compression stockings), medicinal gloves, insecticide treated mosquito – nets **and other prevention, protective medical devices**	X	X			
	**D.TRTM** inhalers, syringes, humidifiers, nebulizers, hot bags, ice packs, first aid kits, bandages **and other treatment devices for personal use**	X	X			
**Inclusion/Exclusion criteria to be specified when asking about the amount** Only applies to some diagnostic products	Includes repair, rental and maintenance					
	**COICOP code 06.1.3 Assistive products for vision, hearing, mobility and daily living.**					
	**A.PURC.VH. Purchase of** glasses for vision; white canes, glass eyes, contact lenses, hearing aids and **other assistive products for vision and hearing**				X	X
	**A.RRMN.VH. Repair, rental/maintenance of assistive health products for vision and hearing**				X	X
	**A.PURC.MD. Purchase of** crutches & wheelchairs; therapeutic footwear; walkers; pressure relief mattresses **and all other assistive health products for mobility and daily living.**				X	X
	**A.RRMN.MD. Repair, rental/maintenance of assistive health products for mobility and daily living.**				X	X
