# Supplementary material for: Effect of specificity of health expenditure questions in the measurement of out-of-pocket health expenditure: evidence from field experimental study in Ghana
Source: BMJ Open. 2021 May 3;11(5):e042562. doi: 10.1136/bmjopen-2020-042562 (PMC8098927; doi:10.1136/bmjopen-2020-042562)
Supplement: Supplementary data [file bmjopen-2020-042562supp003.pdf]

Supplementary material 3: Supplementary tables

Table 1: Demographic Characteristics of households for matched versus unmatched households and households with versus households without OOPs

	Households with any expenditure		Households without any expenditure		Matched Households		Unmatched Households	
Total number of households	N=2093		N=914		N=1300		N=1698	
	n	%	n	%	n	%	n	%
**Sex**								
Male	1332	64	589	64	841	65	1074	63
**Marital status**								
Married	1326	63	519	57	827	64	1013	60
**Level of Education**								
No education	1116	53	474	52	656	50	931	55
Primary	420	20	196	21	262	20	349	21
Junior high school	292	14	128	14	195	15	223	13
Senior high school	97	5	43	5	71	5	70	4
Vocational/Technical/College/Graduate	167	8	73	8	116	9	125	7
**Religion**								
Christians	1114	53	460	50	730	56	841	50
Islam	162	8	81	9	107	8	136	8
Traditional	708	34	326	34	387	30	638	38
No religion	110	5	47	5	76	6	83	5
**Age group**								
15 - 19	96	5	37	4	60	5	74	4
20-34	138	7	58	6	91	7	102	6
35 - 64	1228	59	543	59	781	60	989	58
65 +	630	30	276	30	368	28	533	31
Mean age (SD)	55	17	55	17	54	17	55	17
**Household size**								
1 person	108	**5**	103	**11**	70	**5**	141	**8**
2-5 persons	1010	**48**	516	**56**	616	**47**	906	**53**
6 and above	975	**47**	295	**32**	614	**47**	652	**38**

Table 2: Summary of mean OOPs for unmatched and matched households by spending categories

	Questionnaire Version 1				Questionnaire Version 2				Questionnaire Version-3			
	11 disaggregated health items				44 disaggregated health items				56 disaggregated health items			
		Unmatched Household OOPs		Matched Household OOPs		Unmatched Household OOPs		Matched Household OOPs		Unmatched Household OOPs		Matched Household OOPs
	N	Mean (SD)	N	Mean (SD)	N	Mean (SD)	N	Mean (SD)	N	Mean(SD)	N	Mean (SD)
Outpatient	81	64 (135)	44	66 (128)	181	43 (130)	126	43 (150)	105	44 (78)	47	31 (78)
Inpatient	171	319 (527)	91	226 (279)	177	398 (809)	99	314 (504)	193	287 (716)	100	215 (456)
Medicines	487	41 (140)	302	36 (157)	560	29 (78)	381	25 (56)	609	29 (76)	354	30 (90)
Preventive care	137	59 (95)	80	49 (83)	92	34 (53)	67	31 (53)	46	31 (44)	22	20 (29)
Other medical services	8	203 (201)	2	43 (4)	5	113 (217)	3	15 (10.5)	2	12 (4)	2	12 (4)
Health products	36	71 (133)	19	84 (176)	25	160 (250)	14	178 (289)	18	165 (232)	7	6 (5)

Table 3: Summary of mean OOPs for matched households and provider data by spending categories

	Questionnaire Version 1				Questionnaire Version 2				Questionnaire Version-3			
	11 disaggregated health items				44 disaggregated health items				56 disaggregated health items			
		Provider OOPs	Household OOPs	Estimated ratio (HH/provider) of the means (95% CI)		Provider OOPs	Household OOPs	Estimated ratio (HH/provider) of the means (95%CI)		Provider OOPs	Household OOPs	Estimated ratio (HH/provider) of the means (95%CI)
	N	Mean (SD)	Mean (SD)		N	Mean(SD)	Mean (SD)		N	Mean(SD)	Mean (SD)	
Outpatient	44	34 (46)	66 (128)	1.92	126	22 (52)	43 (150)	1.98	47	14 (15)	31 (78)	2.23
Inpatient	91	49 (74)	226 (279)	6.5	99	48 (97)	314 (504)	6.54	100	21 (38)	215 (456)	10.34
Medicines	302	26 (67)	36 (157)	1.39	381	21 (57)	25 (56)	1.19	354	12 (21)	30 (90)	2.54
Preventive care	80	32 (47)	49 (83)	1.53	67	38 (75)	31 (53)	0.81	22	12 (18)	20 (29)	1.73
Other medical services	2	11 (11)	43 (4)	4.05	3	16 (7)	15 (10.5)	0.94	2	9 (9)	12 (4)	1.33
Health products	19	44 (61)	84 (176)	1.9	14	21 (28)	178 (289)	8.66	7	36 (31)	6 (5)	0.17
