# Supplementary material for: Effect of specificity of health expenditure questions in the measurement of out-of-pocket health expenditure: evidence from field experimental study in Ghana
Source: BMJ Open. 2021 May 3;11(5):e042562. doi: 10.1136/bmjopen-2020-042562 (PMC8098927; doi:10.1136/bmjopen-2020-042562)
Supplement: Author's manuscript [file bmjopen-2020-042562.draft_revisions.pdf]

BMJ Open is committed to open peer review. As part of this commitment we make the peer review history of every article we publish publicly available.

When an article is published we post the peer reviewers' comments and the authors' responses online. We also post the versions of the paper that were used during peer review. These are the versions that the peer review comments apply to.

The versions of the paper that follow are the versions that were submitted during the peer review process. They are not the versions of record or the final published versions. They should not be cited or distributed as the published version of this manuscript.

BMJ Open is an open access journal and the full, final, typeset and author-corrected version of record of the manuscript is available on our site with no access controls, subscription charges or pay-per-view fees (http://bmjopen.bmj.com).

If you have any questions on BMJ Open's open peer review process please email info.bmjopen@bmj.com

BMJ Open

Effect of specificity of health expenditure questions in the measurement of out-of-pocket health expenditure: Evidence from field experimental study in Ghana

Journal:	*BMJ Open*
Manuscript ID	bmjopen-2020-042562
Article Type:	Original research
Date Submitted by the Author:	09-Jul-2020
Complete List of Authors:	Agorinya, Isaiah; University of Health and Allied Sciences, Epidemiology and Biostatistics; Swiss Tropical and Public Health Institute, Epidemiology and Public Health<br>Ross, Amanda; Swiss Tropical and Public Health Institute<br>Flores, Gabriela; World Health Organization<br>TanTorres Edejer, Tessa; World Health Organization, Global Programme on evidence for Health Polic<br>Dalaba, Maxwell Ayindenaba; Navrongo Health Research Centre,<br>Mensah, Nathan; University of Cape Coast, Department of Health Information Management<br>Le My, Lan; Swiss Tropical and Public Health Institute, Epidemiology and Public Health; University of Basel<br>Dessie, Yadeta; Haramaya University, Public Health<br>Sumboh, Jemima; Navrongo Health Research Centre<br>Oduro, Abraham; Navrongo Health Research Centre, Epidemiology<br>Akazili, J; Ghana Health Service<br>Tediosi, Fabrizio; Schweizerisches Tropen- und Public Health-Institut
Keywords:	HEALTH ECONOMICS, EPIDEMIOLOGY, STATISTICS & RESEARCH METHODS

SCHOLARONE™
Manuscripts

I, the Submitting Author has the right to grant and does grant on behalf of all authors of the Work (as defined in the below author licence), an exclusive licence and/or a non-exclusive licence for contributions from authors who are: i) UK Crown employees; ii) where BMJ has agreed a CC-BY licence shall apply, and/or iii) in accordance with the terms applicable for US Federal Government officers or employees acting as part of their official duties; on a worldwide, perpetual, irrevocable, royalty-free basis to BMJ Publishing Group Ltd ("BMJ") its licensees and where the relevant Journal is co-owned by BMJ to the co-owners of the Journal, to publish the Work in this journal and any other BMJ products and to exploit all rights, as set out in our licence.

The Submitting Author accepts and understands that any supply made under these terms is made by BMJ to the Submitting Author unless you are acting as an employee on behalf of your employer or a postgraduate student of an affiliated institution which is paying any applicable article publishing charge ("APC") for Open Access articles. Where the Submitting Author wishes to make the Work available on an Open Access basis (and intends to pay the relevant APC), the terms of reuse of such Open Access shall be governed by a Creative Commons licence – details of these licences and which Creative Commons licence will apply to this Work are set out in our licence referred to above.

Other than as permitted in any relevant BMJ Author's Self Archiving Policies, I confirm this Work has not been accepted for publication elsewhere, is not being considered for publication elsewhere and does not duplicate material already published. I confirm all authors consent to publication of this Work and authorise the granting of this licence.

For peer review only

Effect of specificity of health expenditure questions in the measurement of out-of-pocket health expenditure: Evidence from field experimental study in Ghana

Isaiah Awintuen Agorinya*[1, 2,4,5], Amanda Ross[1,4], Gabriela Flores[6], Tessa Tan-torres Edejer[6], Maxwell Dalaba[3,5], Nathan Kumasenu Mensah[7], Le My Lan[1,4,8], Yadeta Dassie Bacha[9], Jemima Sumboh[3], Abraham Oduro[3], James Akazili[2,3], Fabrizio Tediosi[1,4]

[1]Swiss Tropical and Public Health Institute,
[2]INDEPTH-Network Secretariat, Accra, Ghana
[3]Navrongo Health Research Centre, Navrongo, Ghana
[4]University of Basel, Basel, Switzerland
[5]University of Health and Allied Sciences, Ho, Ghana
[6] World Health Organization (WHO), Geneva, Switzerland
[7]Department of Health Information Management, University of Cape Coast, Ghana
[8]FilaBavi Health and Demographic Surveillance Site, Hanoi, Vietnam
[9]Department of Public Health, College of Health and Medical Sciences, Haramaya University, Harar, Ethiopia

[*] **Corresponding author**

Email: iagorinya@gmail.com (IAA)

For peer review only - http://bmjopen.bmj.com/site/about/guidelines.xhtml

Abstract

Background: The effect of number of health items on OOPs has been identified as a source of bias in measuring OOPs. Evidence comes mostly from cross-sectional comparison of different survey instruments to collect data on OOPs. Very few studies have attempted to validate these questionnaires, or distinguish bias arising from the comprehensiveness of the OOPs list versus specificity of OOPs questions.

Objectives: This study aims to estimates biases arising from the specificity of OOPs questions by comparing provider and household's information.

Methods: A generic questionnaire to collect data on household's OOPs was developed following the nomenclature proposed in division 6 of the COICOP2018. The four categories within such division are used to set the comprehensiveness of the OOPs list, the specificity within each category was tailored to the design of the nationally representative living standard survey in Ghana where a field experiment was conducted to test the validity of different versions. Households were randomized to 11, 44 or 56 health items. Using data from provider records as the gold standard, we compared the mean positive OOPs, and estimated the mean bias and variability in the ratio of household expenditures to provider data for the individual households using the Bland-Altman method of assessing agreement.

Findings: We found evidence of a difference in the overall bias by specificity for OOPs in inpatient care and medications. Within each of these two categories, a more detailed disaggregation yielded lower OOPs estimates than less detailed ones. The level of agreement between household and provider OOPs also decreased with increasing specificity of health items.

Conclusion: Our findings suggest that, for inpatient care and medications, systematically decomposing OOPs categories into finer sub-classes tend to produce lower OOPs estimates. Less detailed items produced more accurate and reliable OOPs estimates in the context of a rural setting.

Keywords

Health expenditure, out-of-pocket payments, universal health coverage, household survey, specificity, validation, Ghana

For peer review only

For peer review only - http://bmjopen.bmj.com/site/about/guidelines.xhtml

Strengths and Limitations of this study

- The main strength of this study is the use of provider data as a 'gold standard' to validate self-reported household out-of-pocket health expenditures in a rural African setting.
- This study has quantified the bias and variability in current household survey instruments that uses different levels of health expenditure items to estimate OOPs.
- The study validated spending categories contained in the latest COICOP revisions (COICOP 2018) which has previously not been done
- Generation of provider data posed as a major limitation to this study as this was not a routine practice by health providers.
- Sample sizes for each spending category was largely affected by the fraction of households reported OOPs that accurately matched with provider data

For peer review only

Introduction

Household out-of-pocket health payments (OOPs) are direct payments for services from households' primary income or savings with no third-party payer involved (SHA, 2011). OOPs are an important measure of performance of the health financing system, particularly to monitor to what extent voluntary, unpredictable and unpooled payments are used to mobilized money within the health system and the impact of such payments on household's living standard and ability to spend on other basic needs (WHO UHC global monitoring report 2019; Xu ke et al. 2018; O'Donnell et al., 2007; O'Donnell, 2019 Thomson et al., 2019; Wagstaff et al., 2018; Xu et al., 2003; Wagstaff et al., 2018).

An important source of information to track OOPs are household surveys, especially in countries where much private health care financing occurs without the generation of linked, reliable and comprehensive routine data. Household consumption and expenditure surveys, household utilization and expenditure surveys, and health surveys with information on health expenditures can be used to gather data on OOPs. None of these surveys are standardized as they can differ, among other things, in the number of main categories covered by the OOPs list as well as the level of details (specificity within each main category of health product or service) (Rannan-Eliya & Lorenzoni, 2010; Lu et al., 2009; Neter & Waksberg, 1964; Neter, 1970; Lavado, Brooks & Hanlon, 2013; Heijink et al., 2011; Xu et al., 2009). Lu (2009) comparing estimates from 50 countries using WHS data reported that, the less comprehensive the questions (corresponding to a smaller number of health categories - 1 category versus 8 categories), the lower the average health spending controlling for other survey characteristics. Heijink (2011) reported from reviewing survey questionnaires for 114 countries that the number of health questions mostly between 1 and 25, with some outliers falling over this range (Heijink et al., 2011). Lavado et al (2013), also found the number of questions on health expenditure to range from 1 to 274 in 214 surveys and estimated that an additional question on OOPs increases health expenditure share by 1% (Lavado, Brooks & Hanlon, 2013). However, neither Lavado et al, nor Heijink et al distinguished bias arising from comprehensiveness of the OOPs list versus specificity within each category. There is a paucity of literature that investigates the accuracy of reported OOPs in household surveys. To the best of our knowledge, no validation study has so far been conducted to assess the effect of the specificity of the OOPs expenditure list on the accuracy and reliability of OOPs statistics in the context of low-middle income countries, let alone rural settings. This paper aims at filling this gap. We compare household responses to provider data in order to assess and quantify agreement. We assess the effect of the specificity of health items within each main category of health product and service on the accuracy of reported OOPs. Specificity here refers to the level of detail in the COICOP health expenditure items list.

For peer review only - http://bmjopen.bmj.com/site/about/guidelines.xhtml

Methods

Strategy

The overall project, in which this study is nested, was implemented in three health and demographic surveillance sites (HDSS) of the INDEPTH-Network located in norther Ghana (Navrongo health and demographic surveillance site, mostly rural), Burkina Faso (Ouagadougou demographic surveillance site, informal urban setting) and Vietnam (FilaBavi demographic surveillance site, mixed rural/urban setting) as part of a project on household out-of-pocket payments (iHOPE). The project aimed to estimate the effect on reported OOPs of the number of health expenditures items within the main categories of health spending and the choice of the recall period for each category of health spending. The INDEPTH-Network platform provided the project with the opportunity to identify and track households and link them to health provider records to be able to validate household reported out-of-pocket expenditures.

In this paper, findings from the study on the effect of the specificity of health expenditure categories on reported OOPs conducted in Navrongo HDSS (Ghana) are presented. For this experiment, three versions of health expenditure modules with different level of specificity of health expenditure categories were developed and adapted to the structure of the Ghana living standards survey 6 (GLSS6) instrument (GLSS6 Report, 2014). These three versions were all comprehensive in that they all captured the major health care consumption groups that constitute the main categories of health care expenditures individuals are confronted with as identified by the classification of household final consumption (COICOP), 2018 version (United Nations, 2018). They differed in the level of detail (specificity) within each class. Mean positive health expenditures were first compared across versions without any gold standard. This is what most studies to date have been able to analyze. The value added of this study is the use of provider records to identify the level of agreement between provider and household records.

Study settings

This study was implemented at the Navrongo HDSS site located in the Northern region of Ghana. The site has two administrative districts with an estimated total population of 160,000. The site has one public hospital, one health research centre, one private clinic, seven health centers, and 27 community-based health compounds. A number of pharmacy shops, chemical and drug shops, petty traders and peddlers, herbalists, faith-based and traditional healers also operate in the area. The research centre collects vital socio-demographic data every four months while household characteristics and assets are collected every two years (Oduro et al., 2012).

For peer review only - http://bmjopen.bmj.com/site/about/guidelines.xhtml

Study design

This study uses health provider records as the ‘gold standard’ to compare to household reported OOPs, recognizing that the provider records are not a perfect gold standard in the absence of a pre-existing formal recording system in place. Two sets of data were collected in this study. The first set of data was captured from households in a cross-sectional field survey conducted between May 2017 and December 2017 and the second set of data was obtained from health provider records. Households were randomized into three versions of a household questionnaire on consumption expenditure for face-to-face interview. All versions of the questionnaire included questions on out-of-pocket health spending for inpatient care services, preventive care services, other outpatient care services, other health services, medicines and health products but differed in the specificity of each one of these OOPs categories. The versions were labeled; version-1 for the questionnaire with 11 items covering the 6 health categories previously mentioned version-2 and 3 for the questionnaire versions with a maximum of 44 and 56 items respectively. All three versions of the instrument used similar recall periods of 4 weeks for items listed under the categories medicines, other outpatient services, other health services; 12 months for inpatient care services and other health products; and 6 months for OOPs related to preventive care services. Supplemental material 1 discusses in detail the structure of the questionnaire. The specificity of OOPs categories is also available from Supplementary material 2.

We created a database of provider records to validate the household reported OOPs. Both private and public health care providers within the study area were engaged by the project team to extract records covering a 13 month period which in some cases (mostly for private providers) also required improving the recording of provider records. Health expenditures reported by any member of the household were tracked and a corresponding health provider record obtained to create a matched dataset (referred to as the matched data) for validation. Household heads were the most common respondents for the household survey but in some cases, other individuals within the household were nominated by the household head to provide responses.

Study population and Sampling

All households registered under the Navrongo demographic surveillance site database constituted our study population. The households were randomly selected. The sample size for the household cross-sectional survey was based on estimating the agreement between two quantitative measurements. We followed the Bland and Altman approach which suggests a rule of thumb that 100-200 observations is adequate for sufficient precision when assessing agreement (Bland, 2004). Based on unpublished district health management report in the study area, the probability of spending on outpatient for a 2-week recall period was 15.5% and 10% for a 12-month recall for inpatient care. To achieve a minimum of 100 households with reported inpatient care (outpatient spending is included in this sample), the sample size required would be 1000 households plus 10%

For peer review only - http://bmjopen.bmj.com/site/about/guidelines.xhtml

non-response rate which gives a total of 1100 households per questionnaire version, and 3300 households for all three versions.

Health financing system in Ghana

Ghana is one of very few countries to have enacted a legislation (National Health Insurance Act 2003 (Act 650) and begun the transition to universal health insurance coverage (National Health Insurance Scheme, NHIS) to replace the OOPs previously referred to as "Cash and Carry" system. The scheme is largely financed through tax and a small proportion from - contributions and donations. In 2014, the scheme covered only about 40% of Ghana's population (10.5 million active subscribers) with about 69% of these exempted from any form of payment to the scheme (Wang, Otoo & Dsane-Selby, 2017) . The exempt groups include indigent people, pregnant women and very poor households covered by a social intervention programme called Livelihood Empowerment Against Poverty (LEAP). The National Health Insurance Scheme covers 95% of disease conditions reported in Ghana with services including primary curative care to care at tertiary facilities but challenges within the operationalization of the scheme are thought to expose subscribers to OOPs (addae-korankye, 2013). All subscribers accessing health care from NHIS accredited health facilities are assured of free services but may be exposed to spending out-of-pocket for medicines, laboratory tests and other consumables which may not be available at the provider due for example to stock-outs (Addae-korankye (2013)). The uninsured population (accounting for about 60% in 2014) will be required to pay out-of-pocket to be able to access health care (Wang, Otoo & Dsane-Selby, 2017) . Therefore, we expect some level of OOPs especially for medicines and hospitalization in the study area.

Data analysis

We carried out separate analyses for the matched and unmatched data to assess the effect of the number of health items on the accuracy of the reported OOPs and the reported OOPs respectively. We assess the consistency of the findings in these two analyses. For the unmatched samples, we summarized the OOPs by spending categories and household characteristics for each questionnaire version using arithmetic means and standard deviations for continuous variables and proportions for categorical variables. This is consistent with the methodological approach used in most papers to date without information from the provider. We summarized the effect of number of items on mean reported OOPs by spending category using the ratio of each category to the reference version (verstion-1:11 items). The confidence intervals for the ratio were calculated using bootstrapping method. Only four categories of spending were considered instead of six due to the small number of households reporting any OOPs for health products and other health services (Table 3).

For the matched samples, we adopted the Bland-Altman approach (Giavarina, 2015; Bland & Altman, 1999) to assess the level of agreement between household reported OOPs and corresponding provider records for

For peer review only - http://bmjopen.bmj.com/site/about/guidelines.xhtml

For peer review only

each questionnaire version separately. We calculated the ratio of individual household to matched provider OOPs. The ratio was preferred to the simple difference since it had a reasonably constant distribution over the range of the provider OOPs, whereas the difference between household and provider OOPs was dependent on the level of provider OOPs. The log-transformation of the ratio was used for analysis due to the skewed distribution of the ratios (Giavarina, 2015; Bland & Altman, 1999). For each questionnaire version, we estimated the overall mean bias using the geometric mean of the ratios and the variability using the 95% limits of agreement (LoA) (i.e. limits within which 95% of the ratios are expected to lie). To compare the three versions of the questionnaire and assess the effect of number of health items on OOPs, we compared the overall mean bias and variability using regression models proposed by Bland-Altman (Bland & Altman, 1999). Specifically, for the mean bias, we fitted a regression model with the log ratio of household to provider OOPs as the outcome variable and questionnaire version as an explanatory variable. This provides an estimate of the effect of the questionnaire version on the bias, measured by the mean log ratio with corresponding confidence interval and p-value. We included a random effect parameter to account for the clustering of the households within clusters defined by the Navrongo HDSS. For the effect of the questionnaire version on the variability, a second regression model was used with the outcome of the absolute values of the residuals previously obtained against the questionnaire version. Since the dependent variable in the first regression is on the log scale, all results were transformed to the ratio scale for ease of interpretation. Questionnaire version 1 which has the least number of health items (11 items) was used as the reference group for the regression models. Data was analyzed using STATA Version 14, Stata Corp.

Ethics approval and consent to participate

The Ethical Review Board of the Navrongo Health Research Centre, Ghana (NHRCIRB217) approved for the conduct of the study. Informed consent was obtained from all study participants.

Patient and Public Involvement

Per the design and objective of this study, it was not appropriate to involve the public in the design, or conduct, or reporting, or dissemination plans of our research.

For peer review only - http://bmjopen.bmj.com/site/about/guidelines.xhtml

Results

Sample distribution and matching

A total of 3300 households were sampled and randomized to receive the three questionnaire versions. The response rate was 83% in version 1, 94% in version 2 and 94% in version 3. Approximately 70% of households reported any form of OOP across all three questionnaire versions as expected. In terms of the matching of household expenditures to provider data, the proportion of households that matched was 60% in version 1, 65% in version 2 and 55% in version 3. Figure 1 below shows a flow chart that summarizes the sample distribution, the proportion of households incurring OOPs and the overall proportion of OOPs that matched with provider records in each questionnaire version.

Figure 1: flow chart of household enrolment and matching to provider records

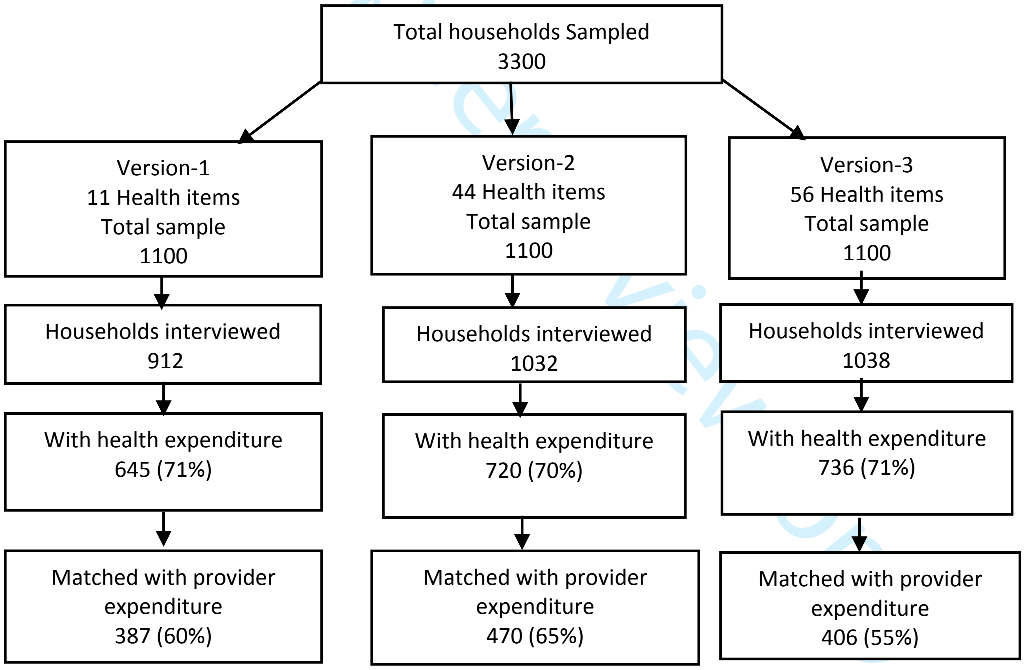

For peer review only - http://bmjopen.bmj.com/site/about/guidelines.xhtml

Socio-demographic characteristics of households

A total of 3023 households were interviewed out of 3300 sampled. Of these, 64% of the household heads were males, the majority (89%) of the household heads were above 34 years of age, 62% of the household heads were married, slightly more than half (53%) of household heads did not have any formal education or were Christians and the mean age was 55 years (17 SD). The majority of households had more than one member with 51% having 2-5, and 42% with 6 or more (Table 1). From Table 1 in supplementary material 3, it can be seen that, the distribution of demographic characteristics was similar between households in the three questionnaire versions, households with health expenditure versus those without, and households with matched provider records versus households without successful matched provider records except for household size where the proportions differed significantly by version for household size great than 1and less than 6 (Supplementary material 3: Table 1). We however do not expect this difference in proportion to drive the differences in the household reported OOPs since the differences are drastically reduced if we considered household size of one versus those more than one.

For peer review only

Table 1: Demographic Characteristics of household and household head

	Questionnaire version 1		Questionnaire version 2		Questionnaire version 3		All Questionnaire versions combined	
Total number of households	N=925		N=1062		N=1036		N=3023	
	n	%	n	%	n	%	n	(%)
**Gender**								
Male	582	63	705	66	647	62	1934	64
**Marital status**								
Married	566	61	687	65	606	59	1859	62
**Level of Education**								
No education	451	49	574	54	572	55	1597	53
Primary	200	22	214	20	202	20	616	20
Junior high school	144	16	141	13	136	13	421	14
Senior high school	44	5	51	5	49	5	144	5
Vocational/Technical/College/ Graduate	86	9	82	8	77	7	245	8
**Religion**								
Christians	519	56	501	47	571	55	1591	53
Islam	54	6	148	14	41	4	243	8
Traditional	314	34	355	33	362	35	1031	34
No religion	38	4	58	6	62	6	158	5
**Age group**								
15 - 19	40	4	56	5	43	4	139	5
20-34	51	6	77	7	67	7	195	6
35 - 64	572	62	621	59	589	57	1782	59
65 +	262	28	308	29	337	33	907	30
Mean age (SD)	55	17	54	17	56	17	55	17
**Household size**								
1 person	67	7	82	8	64	6	213	7
2-5 persons	418	45	549	52	566	55	1533	51
6 and above	441	48	432	41	403	39	1276	42

For peer review only - http://bmjopen.bmj.com/site/about/guidelines.xhtml

Probability of reporting any OOPs by spending category

Overall, 2969 households provided information about household health spending of which 71% of households reported any form of OOPs. The proportion of all households reporting OOPs for inpatient care over the 12 month recall period was 18%, for preventive care over the past 6 month recall period 9%, for outpatient care within the past 4 weeks 12%, and slightly more than half (56%) of the households also reported having incurred OOPs on medications over the past 4 weeks regardless of the number of health items (Table 2).

Table 2: Health expenditures – Proportion of households reporting positive OOPs by spending category

	Questionnaire version-1			Questionnaire version -2			Questionnaire version -3			Overall	
	N=901			N=1032			N=1036			N=2969	
number of households with positive health by category: new COICOP classification	Number of health items	n	%	Number of health items	n	%	Number of health items	n	%	n	%
inpatient care services	2	170	19	14	177	17	14	193	19	540	18
preventive services	2	137	15	5	92	9	5	46	4	275	9
Other health services	1	9	1	2	5	0.5	2	2	0.2	16	0.5
outpatient	2	81	9	12	181	18	12	105	10	367	12
medicines	2	487	54	9	560	54	16	609	59	1656	56
health products	2	36	4	2	25	2	7	18	2	76	3
number of households with any health expenditure	11	645	71	44	720	70	56	736	71	2101	71

Mean OOPs reported by households by spending category (unmatched analysis)

The mean household OOPs tended to be larger for lower number of health expenditure items (less specificity) in all main spending categories except health products where we see the ratio of the means to be greater than 1, and inpatient care where there was no consistent pattern with increasing specificity. The differences in specificity between questionnaire versions were significant for only preventive care and medicines (Table 3). Table 2 and 3 in Supplementary material 3 also shows the summary of mean OOPs compared for matched and unmatched households versus provider estimates across the three versions and by spending categories. The tables show that, there are similarities in average OOPs between matched and unmatched households in inpatient care, medicines and outpatient care. The results also show that household OOPs are on average higher than corresponding provider OOPs.

For peer review only - http://bmjopen.bmj.com/site/about/guidelines.xhtml

Table 3: Arithmetic mean OOPs by health category and questionnaire version

	Questionnaire Version-1				Questionnaire Version-2					Questionnaire Version-3				
	11 disaggregated health items				44 disaggregated health items					56 disaggregated health items				
Spending category	Number of health items aggregated	N	household average OOPs (Ghc)	SD	Number of health items aggregated	N	household average OOPs (Ghc)	SD	Estimated ratio of the means (Version-2/version-1) (95%CI)	Number of health items aggregated	N	household average OOPs (Ghc)	SD	Estimated ratio of the means (Version-3/version-1) (95%CI)
outpatient	2	81	64	135	12	181	43	130	0.70 (0.20 – 1.21)	12	105	44	78	0.75 (0.27 – 1.22)
inpatient	2	171	319	527	14	177	398	809	1.25 (0.75 – 1.74)	14	193	287	716	0.92 (0.51 – 1.34)
medicines	2	487	41	140	9	560	29	78	0.71 (0.44 – 0.98)	16	609	29	76	0.66 (0.44 – 0.88)
Preventive care	2	137	59	95	5	92	34	53	0.60 (0.33 – 0.87)	5	46	31	44	0.57 (0.27 – 0.88)
Other medical services	1	8	203	201	2	5	113	217	0.56 (-)	2	2	12	4	0.06 (-)
Health products	2	36	71	133	2	25	160	250	2.38 (-)	7	18	165	232	2.32 (-)

Note: the currency used is the Ghana cedi (GHC). US GHc4.2 was equivalent to US1$ at the time of collecting data.

Quantifying the mean bias and variability between household and provider OOPs (matched analysis)

We assessed the agreement between the matched household and provider OOPs first for each questionnaire version separately by estimating the overall bias and variability for each type of health expenditure category. The overall bias, measured as the geometric mean ratio of household to provider OOPs, increases with increasing numbers of health items for OOPs in inpatient care and medicines (Table 4). There was no evidence of an effect for outpatient or preventive care. This evidence suggests that disaggregating health expenditure items into finer specific items tended to decrease the level of agreement between matched household OOPs and corresponding provider records for inpatient care and medicines.

For peer review only

For peer review only - http://bmjopen.bmj.com/site/about/guidelines.xhtml

Table 4 Mean bias and variability in measurement of OOPs by number of health expenditure items by spending category

	Number of households	Mean bias	95% limits of agreement of mean bias	Estimated difference in mean bias between questionnaire versions & CI & p-value	Estimated diffference in SD of mean bias between questionnaire versions & CI & p-value
Outpatient care				p = 0.49	p = 0.50
2 health items	44	1.02	0.05 – 21.2	-	-
12 health items	126	1.21	0.05 – 26.7	1.29 (0.74 – 2.23)	1.02 (0.73 – 1.42)
12 health items	47	1.56	0.12 – 19.6	1.47 (0.77 – 2.80)	0.84 (0.57 – 1.25)
Inpatient care				p = 0.003	p = 0.01
2 health items	91	3.88	0.17 – 86.2	-	-
14 health items	99	6.61	0.16 – 270.7	1.63 (0.99 – 2.69)	1.35 (1.03 – 1.76)
14 health items	100	9.19	0.51 – 161.2	2.34 (1.44 – 3.83)	0.93 (0.71 - 1.21)
Medicines				p = 0.023	p = 0.33
2 health items	302	1.26	0.10 – 16.0	-	-
9 health items	381	1.35	0.09 – 19.5	1.14 (0.91 – 1.42)	1.04 (0.91 – 1.19)
16 health items	354	1.62	0.18 – 15.0	1.36 (1.01 – 1.70)	0.95 (0.83 – 1.08)
Preventive care				p = 0.290	p = 0.51
2 health items	86	1.21	0.09 – 15.15	-	-
5 health items	67	0.89	0.04 – 18.9	0.74 (0.46 – 1.14)	1.19 (0.88 – 1.62)
5 health items	22	1.33	0.08 – 22.4	1.07 (0.55 – 2.05)	1.04 (0.68 – 1.60)

Note: The unit of the estimated difference is the mean bias expressed as a ratio between Household and provider for each questionnaire version

We investigated why the unmatched analysis pointed to lower mean OOPs with increasing numbers of items, but the matched analysis to higher mean OOPs. There were variations in the proportions of households with matched records across type of service received by household members and type of health providers. OOPs incurred from diagnostics, the pharmacy and community health and family planning service facilities (CHPS zones) patient tended to match better than in the hospitals as observed in Table 2 of supplementary material 1. The demographic characteristics of households were similar for all households with OOPs and the matched households only (Supplementary material 3 Table 1). The mean OOPs within spending categories tended to be lower for the matched households (Supplementary information 3 Table 2). The moderate matching rates, and a tendency for higher household-reported OOPs to be omitted, suggests that the matching process may have influenced the matched analysis results.

For peer review only - http://bmjopen.bmj.com/site/about/guidelines.xhtml

Discussion

We present evidence of the influence of the specificity of health categories on the bias and variability of reported household positive OOPs by spending category. This is done by comparing household and provider data. In this study, we found that the unmatched analysis (where household data is not compared with provider data), comparing mean reported OOPs for all households, suggested a tendency for a higher number of items to result in lower positive OOPs for medicines and preventing services. This suggests that being more detailed as a consequence of increasing the number of health items within each category leads to lower mean positive OOPs in these spending categories. In assessing the agreement between matched household-reported and provider OOPs, our findings suggests that, the level of agreement between provider and household OOPs decreased with increasing specificity for medicines and inpatient care. These trends maybe attributed to the inability of households to either recall the specific name of the medicine/service received from the health provider or they did not know the name of the medicine/service at all when such information is required at a very detailed level. Another potential factor could be a possible introduction of bias by the matching process in our study, with the possibility that smaller amounts matched better with provider data than larger amounts. These factors may require further investigations beyond the scope of this current study, however, another yet to publish paper from the iHOPE project has offered some useful context for this.

A number of studies have investigated and documented the potential effect and consequence of varying number of health items on the estimation of OOPs using nationally representative survey data (Lu et al., 2009; Lavado, Brooks & Hanlon, 2013; Xu et al., 2009; Grosh & Glewwe). All these studies have mostly focused their investigations on the effect of number of health items on total household health expenditure without examining the effect by spending categories or distinguishing between comprehensiveness of the health expenditure list (number of health categories covered) from specificity of each category (number of questions per health category) therefore making such studies deficient in identifying the most reliable and accurate survey tools.

Our findings complement such literature by focusing more on the effect of specificity on mean bias and variability in these survey instruments by controlling for possible confounders across different versions such as changes in recall period and relying on provider data to compare across versions varying in the level of detail for each health category. This study employed a field experimental study approach, where more and less detailed versions of a questionnaire on OOPs are tested, and we find that the less disaggregated version provides the more accurate information when compared to provide records in particular for inpatient care and medicines. The focus has been on inpatient and medicines because they constituted the major OOPs spending categories in the study location. This new information provides a context to be able to identify existing household survey instruments that provide relatively more accurate information on household OOPs.

For peer review only - http://bmjopen.bmj.com/site/about/guidelines.xhtml

The questionnaire is implemented in the context of rural Northern Ghana where people are covered for their direct inpatient expenditures by insurance but non-the-less, challenges (addae-korankye, 2013) within the insurance system still exposes care seekers to OOPs especially for inpatient care and medicines. Within this context of rural settings, the use of provider data presented as a major limitation for the study. Health providers were not routinely collecting patient/client data. This resulted in incomplete and potentially inaccurate generation of data by some of the health providers. This influenced the proportion of households that matched with provider data and subsequently influenced how our results are interpreted. Despite these limitations, our study has provided some context that will guide household survey designers in framing questions.

We also acknowledge that, our findings may be different in other context and therefore similar field experimental studies are being conducted in urban Burkina Faso and urban/rural areas of Vietnam as part of the iHOPE project in an attempt to generalize results to be able to appropriately identify valid, reliable and comparable methods for estimating OOPs in household surveys.

Conclusion

This study describes a novel validation study in a rural setting that operates a demographic surveillance system. The evidence from this study suggests caution in interpreting OOPs from different survey instruments relying on different levels of detail per spending category even in the absence of differences in recall period and other survey questionnaire design features. We found that systematically decomposing health expenditure items into more specific and finer sub-classes leads to lower average OOPs for outpatient care, medicines and preventive care when comparing across different version without comparing to provider records. Validation studies need to consider the possibility of bias introduced by the matching approaches.

For peer review only

List of abbreviations

DHS Demographic and Health Survey

NHRC Navrongo Health Research Centre

NHDSS Navrongo Health and Demographic Surveillance System

GLSS Ghana Living Standards Survey

COICOP Classification of Individual Consumption according to purpose.

iHOPE INDEPTH-Network Household Out-of-pocket Expenditure

HHS Household Health Survey

SHA System of Health Accounts

NHA National Health Accounts

NHIS National Health Insurance Scheme

OOPs Out-of-Pocket Health Spending

LMIC Low and Middle-income Countries

LSMS Living Standards Measurement Survey

WHO World Health Organization

WHS World Health Survey

Consent for publication

Written informed consent was obtained from all patients for publication of this manuscript.

Availability of data and material

The data is not publicly available. However, upon reasonable request, the data will be available from the corresponding author.

Competing interests

The authors declare that they have no competing interests.

Funding

This project was jointly funded by the INDEPTH-Network in Accra and the Swiss tropical and public health institute of the University Of Basel, Switzerland through a grand from Bill and Melinda gates foundation, grant number OPP1113162. GF, TE, AR were partially supported by WHO.

For peer review only

For peer review only - http://bmjopen.bmj.com/site/about/guidelines.xhtml

Author contributions

The research protocol was developed by TE, GF, JA, FT

The research protocol was written by GF, TE, JA, FT, YDB

The questionnaires were developed by GF, IAA

The analytical plan was developed by AR, IAA, LML, GF

Data collection: IAA, MD, NM

Data cleaning: IAA, NM, MD, JS

Data analysis: IAA, AR

First draft was written by: IAA, AR

All authors contributed to writing of the manuscript

Acknowledgements

The authors wish to thank all the study participants, health facilities and the entire iHOPE Ghana team for participating in this study. We are very grateful to INDEPTH-Network and Swiss Topical and Public Health Institute for their technical support during the entire phase of this study. We also appreciate the financial support from the Bill and Melinda Gates foundation for the study.

For peer review only

For peer review only - http://bmjopen.bmj.com/site/about/guidelines.xhtml

References

Bland JM (2004). Sample size for a study of agreement between two methods of measurement [web site]. (https://www-users.york.ac.uk/~mb55/meas/sizemeth.htm, accessed 1 March 2019).

Bland JM, Altman DG (1999). Measuring agreement in method comparison studies. , (Statistical Methods in Medical Research):26.

Giavarina D (2015). Understanding Bland Altman analysis. *Biochemia Medica*, 25(2):141–151.

GLSS6 Report (2014). *Ghana Living Standards Survey Round 6 (GLSS6) Main Report*. Ghana Statistical Service,:244 (http://www.statsghana.gov.gh/gssmain/fileUpload/Living%20conditions/GLSS6_Main%20Report.pdf, accessed 11 October 2019).

Grosh EM, Glewwe P. Designing Household Survey Questionnaires for Developing Countries: Lessons from Ten Years of LSMS Experience. :78.

Heijink R et al. (2011). Validity and Comparability of Out-of-pocket Health Expenditure from Household Surveys: A review of the literature and current survey instruments. (https://www.who.int/health_financing/documents/dp_e_11_01-oop_errors.pdf?ua=1, accessed 25 February 2019).

addae-korankye alex (2013). Challenges of Financing Health Care in Ghana: The Case of National Health Insurance Scheme (NHIS).

Lavado RF, Brooks BP, Hanlon M (2013). Estimating health expenditure shares from household surveys. *Bulletin of the World Health Organization*, 91(7):519-524C.

Lu C et al. (2009). Limitations of methods for measuring out-of-pocket and catastrophic private health expenditures. *Bulletin of the World Health Organization*, 87(3):238–244.

Neter J (1970). Measurement Errors in Reports of Consumer Expenditures. *Journal of Marketing Research*, 7(1):11–25.

Neter J, Waksberg J (1964). A Study of Response Errors in Expenditures Data from Household Interviews. *Journal of the American Statistical Association*, 59(305):18–55.

O'Donnell O et al. (2007). *Analyzing Health Equity Using Household Survey Data: A Guide to Techniques and their Implementation*. The World Bank (http://elibrary.worldbank.org/doi/book/10.1596/978-0-8213-6933-3, accessed 27 February 2019).

O'Donnell O (2019). Financial protection against medical expense. *Tinbergen Institute Discussion Paper*:47.

Oduro AR et al. (2012). Profile of the Navrongo Health and Demographic Surveillance System. *International Journal of Epidemiology*, 41(4):968–976.

Rannan-Eliya RP, Lorenzoni L (2010). *Guidelines for Improving the Comparability and Availability of Private Health Expenditures Under the System of Health Accounts Framework*. (https://www.oecd-

For peer review only - http://bmjopen.bmj.com/site/about/guidelines.xhtml

ilibrary.org/social-issues-migration-health/guidelines-for-improving-the-comparability-and-availability-of-private-health-expenditures-under-the-system-of-health-accounts-framework_5kmbrcg0clvc-en, accessed 25 February 2019).

SHA (2011). *A System of Health Accounts*. OECD Publishing (http://www.oecd-ilibrary.org/social-issues-migration-health/a-system-of-health-accounts_9789264116016-en, accessed 12 July 2017).

Thomson S et al. (2019). *Can people afford to pay for health care? new evidence on financial protection in Europe: [regional report*. Copenhagen, World Health Organization, Regional Office for Europe (https://apps.who.int/iris/bitstream/handle/10665/311654/9789289054058-eng.pdf?sequence=1&isAllowed=y, accessed 14 October 2019).

United Nations (2018). Classification of Individual Consumption According to Purpose (COICOP) 2018. *United Nations*:265. (Statistical papers).

Wagstaff A et al. (2018). Progress on impoverishing health spending in 122 countries: a retrospective observational study. *The Lancet Global Health*, 6(2):e180–e192.

Wang H, Otoo N, Dsane-Selby L (2017). Ghana National Health Insurance Scheme. :71.

Xu K et al. (2003). Household catastrophic health expenditure: a multicountry analysis. *The Lancet*, 362(9378):111–117.

Xu K et al. (2009). Assessing the reliability of household expenditure data: Results of the World Health Survey. *Health Policy*, 91(3):297–305.

For peer review only - http://bmjopen.bmj.com/site/about/guidelines.xhtml

Supplementary material 1

Data collection instrument design and matching procedure

Ghana Living Standards Survey 6 (GLSS6)

The Ghana Living Standards survey (GLSS) is a multi-purpose household survey instituted by the World Bank to collects information on many different dimensions of living conditions, including education, health, employment and household expenditure on food and non-food items. The GLSS 6 is the sixth edition of the survey conducted in 2012/2013 (GLSS6 Report, 2014). The survey instrument has one large module on household consumption reported by a single respondent and structured around food versus non-food expenditures (health expenditure included) using similar recall periods for all food and non-food divisions regardless of their relevance in gathering information on health. The consumption module in the survey is comprehensive in covering household consumption items. The structure of the consumption module follows the Classification of individual Consumption According to Purpose (COICOP 1999) classifications. COICOP-1999 is the international reference classification of household expenditure which aims at providing a framework of homogeneous categories of goods and services considered a function or purpose of household consumption expenditure. Following this classification, the GLSS6 has 251 items in the consumption module, of these; about 7% (17 items) are questions asking about health expenditure. The GLSS6 adopted the second level of disaggregation of COICOP-1999 for collecting information on Health expenditure in the consumption module but COICOP-1999 was of little guidance when there is an interest in refining the information on health expenditure beyond the second level of disaggregation. Previous analysis (Lavado, Brooks & Hanlon, 2013) has shown that as the total number of health and non-health consumption expenditure question increases, the average health expenditure budget share tends to either decrease or increase and as such given the structure of the GLSS, it is difficult to assess the accuracy of the information collected. The GLSS has been the primary source of data for estimating OOPs in Ghana.

Specificity of the iHOPE household survey instrument

The starting point to develop questions on OOPs was the GLSS6 but with the objective to align it with division 6 of COICOP-2018 which was refined to better cover all the different type of health products and services available to the population. Version 1 of the OOPs questionnaire captured six major categories namely medicines, health products, preventive care services, other outpatient care services, inpatient care services and other health services to obtain a total of 11 health items. The second questionnaire further expanded all main six categories (Table 1) to a total of 44 health items. In the third questionnaire version only questions on OOPs for medicines and health products were expanded to arrive at a total of 56 health items for version three. Main difference between questions in OOPs included in the GLSS6 and those developed for this study are listed in Table 1.

For peer review only - http://bmjopen.bmj.com/site/about/guidelines.xhtml

The new developed questions on out-of-pocket health care expenditure were then included at the end of GLSS6 consumption module. Questions related to the provider were asked to each person reporting an OOP in order to be able to compare provider and household information. The final instruments included in addition to the consumption expenditure module a household roster, a module on housing characteristics and household assets. Figure 1 shows the generic structure of the developed instrument.

Figure 1: Structure of survey instruments

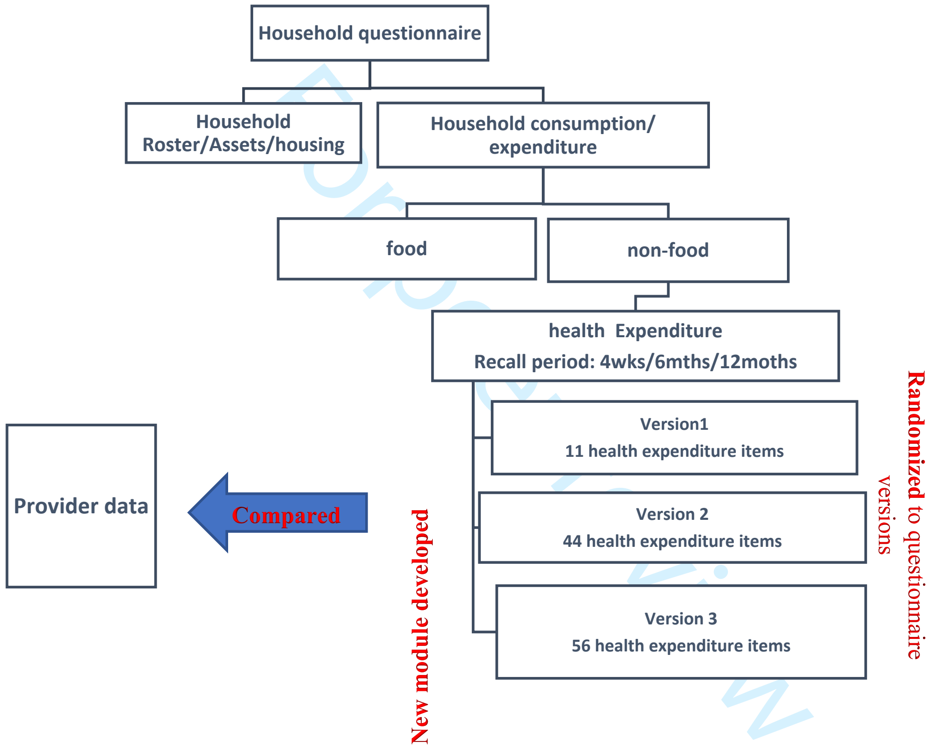

Table 1: Specificity of the health expenditure list

Table 1GLSS 6	Version 1	Version 2	Version 3
Based on COICOP-1999, cover all major classes of health products and services.	covers all major classes of health products and services are redefined in COICOP-2018		
Total number of questions on OOPs for health is 24 split as follows:	Total number of questions on OOPs for health is 11 split as follows:	Total number of questions on OOPs for health is 44. Expand version 1 as follows:	Total number of questions on OOPs for health is 56. Expand version 2 as follows:
Pharmaceutic products and other medical products – 6 questions	Medicines – 2 questions to distinguish herbal from non-herbal medicine	Expands questions on non-herbal medicines (7 questions)	Questions on non-herbal medicines are split into prescribed and non-prescribed medicines
MEDICAL PRODUCTS, APPLIANCES AND EQUIPMENT – 2 questions to differentiate appliance and equipment from assistive products	Health products – 2 questions to distinguish assistive products from an overall category on medical diagnostic products, prevention and protective devices.	Differentiates between purchase of assistive products and repair/rental/maintenance of assistive products	Within purchase and repair of assistive products a distinction is made between those for vision and hearing versus mobility and daily living. The overall category on medical diagnostic products, prevention and protective devices is split into 3.
Outpatient medical services – 14 questions to differential type of health care provider and public/private	Outpatient medical services – 4 questions to differentiate within preventive services immunization/vaccination services from other preventive services (e.g. check-ups); dental outpatient services from other outpatient services.	Immunization vaccination services differentiate between those related to maternal and child care from other vaccination services.<br><br>Other preventive services are split into 3 categories<br>Dental care services and other outpatient services are split by type of facility (hospital versus outpatient setting) and OOPs for consultation are differentiated from OOPs for laboratory tests, imagining services when possible. In addition for other outpatient services the type of health care provider is also distinguished (e.g. specialist, general doctor)	
Hospital services – 2 questions to distinguish public/private	Inpatient services – 2 questions to differentiate long term inpatient care from other overnight stays	Details on inpatient long term care and other overnight stays are gathered by the means of	
	Other health services - 1 question to capture emergency transportation and emergency rescue services	Emergency transportation is split into 2 categories 7 questions covering fees, products, tests and emergency transportation related to the inpatient treatment	

Provider data collections

Within the Ghana health care system, public health providers who are managed by the government keep patient records as part of routine activities while most private providers either kept minimal transactional records or no records at all. To generate accurate data for comparison, we developed a template (Supplementary material 2) and trained private provider owners on how to use it to collect patient information. The main fields in the template included name, address, phone number, referral status, reason for consultation and cost of treatment/service. For public providers where patient data was already

recorded, trained field workers completed the template by review and extracting relevant information from the provider records. We therefore purposely selected major health care providers within the demographic surveillance site to obtain the provider data. The criterion for selecting the providers was based on the availability of transactional data or a care-taker who could record details of the transactions from clients in the case of private providers. The public health providers selected include; one hospital, one clinic and seven public health centres. For the private health care providers, ten high volume pharmacy and license chemical shops met our selection criteria and were selected.

Matching

Matched samples in this study refer to households that were linked to their provider records. For any households that reported positive expenditure on any of the health expenditure items, corresponding health provider data was obtained from the provider records using details about the provider obtained from the respondents. The linked household-provider data formed a matched sample used in our analysis. The matching of household and provider data was done at the individual household member level and by spending category. Due to challenges of completeness and accuracy of provider details provided by respondents and possible errors in recording patient details at the provider, some households could not be matched to provider data and were therefore excluded from the analysis. The challenges that influenced the completeness and accuracy of provider data are discussed in another paper "Challenges in linking household health expenditure records to provider records" (Agorinya et al, in prep)

Table 2: OOPs that successfully matched with health provider records by type of health provider

Type of provider	Total number of clients attending provider	proportion of clients with linked records to household
Hospital	453	46.8
Community Health Centre	195	55.4
CHPS	196	90.3
Clinic	58	27.6
Chemical Shop	194	71.1
Diagnostic laboratory	29	82.8
Hawker/Vendor/ Mobile Van	25	0.0
General local shop	81	33.3
Other	16	12.5
Pharmacy	155	73.6
Total	1402	59

For peer review only

Table 3: OOPs that successfully matched with health provider records by type of care received

Spending category	Total number of cases	Number of cases matched	Proportion of cases matched
Inpatient care	339	159	47.2
Out-patient	551	351	63.4
Medicines	468	286	61.6
Preventive care	32	22	68.8
Medical products	7	1	14.3
Total	1397	820	59

For peer review only

Supplementary material 2

COICOP 2018 based Levels of disaggregation of Health items

Type of information	*Version-1*<br>**Level of *D*isaggregation – 11 Health items**	Recall periods: D- days/ M-Months				
		15d	30d	3M	6M	12M
	**COICOP code 06.2.3 Inpatient care services**					
**To be asked in bold**<br>Examples are given in brackets for more see explanatory notes.<br>**If helpful can add at the end of the explanations: for patients with disabilities, the elderly (or those who requires permanent surveillance or constant help due to limited functional capacity)**	**I.LONG. Medical treatment and / or care that required overnight stay in a nursing home; (medical convalescent homes; palliative care establishments) or any other long term care medical facility**				X	X
	**I.CURR. Medical and dental treatment that required an overnight stay in any type of facility** (e.g. hospitals, clinics) **excluding long term care medical facility**				X	X
**Inclusion/Exclusion criteria to be specified when asking about the amount**<br>Applies to I.LONG & I.CURR	Includes payments for all medical services, diagnostic and laboratory tests, medicines and medical products needed during the overnight stay. Also include emergency transportation services and emergency rescue.<br>Excludes: non-emergency transportation and non-medical costs for patient's relative.					
	**COICOP code 06.2.1 Preventive care services**					
	**P.IMMV. Immunization/vaccination services including for maternal and child care**			X	X	
**Inclusion/Exclusion criteria to be specified when asking about the amount**	Includes ; travel and tourism vaccination as well as any other immunization/vaccination service.					
	**P.OTHR. Other preventive services such as prenatal/postnatal care, child growth and development visits, family planning, screening, tests, consultations to detect communicable or non-communicable diseases before symptoms appear** (e.g. diabetes, heart problems)			X	X	
**Inclusion/Exclusion criteria to be specified when asking about the amount**<br>Applies to both P.IMMV & P.OTHR	Includes diagnostic and laboratory tests needed to provide preventive services but exclude payments for the vaccine itself when separately invoiced from the service.					
	**COICOP code 06.4.2 Emergency transportation and emergency rescue**					
**Alternative wording: transportation for medical emergency reasons (e.g. by ambulance)**	**O.EMER. Patient emergency transportation services and emergency rescue services (excluding those associated with an overnight stay)**	X	X			
**Inclusion/Exclusion criteria to be specified when asking about the amount**	Excludes non-emergency transportation services					
	**COICOP code 06.2.2 & 06.2.3 Outpatient dental & other outpatient services**					
	**O.DENT. Dental consultations and services that did not require an overnight stay;**	X	X			
**Inclusion/Exclusion criteria to be specified when asking about the amount**	Includes dental diagnostics services and laboratory tests needed to provide outpatient dental services (e.g. X-rays, blood tests)<br>For any dental illness, disease, injury or health problem; from any type of provider; inside or outside a hospital setting					
	**O.CRRL. other medical consultations and services than dental and preventive that did not require an overnight stay**	X	X			
**Inclusion/Exclusion criteria to be specified when asking about the amount**	Includes any diagnostic and laboratory test needed to provide outpatient medical services (e.g. X-rays, blood/urine tests), but excludes emergency transportation services and emergency rescue					
	**COICOP code 06.1.1**					
	**M.HERH. Herbal medicines (tablets or syrups) and homeopathic products for consumption outside a health facility or institution.**	X	X			
	**M.MVCP. Medicines** (branded, generic)**, vaccines, oral contraceptives, vitamins and minerals and other pharmaceutical preparations for consumption outside a health facility or institution.**	X	X			
	**COICOP code 06.1. 2 medical diagnostic products, prevention and protective devices**					
**Inclusion/Exclusion criteria to be specified when asking about the amount**	**D.** (Pregnancy tests, incontinence products and absorbent including diapers for the aging population, inhalers, mechanical contraceptives; insecticide treated mosquito nets, blood pressure devices) **and other medical health products for personal use outside a health facility or institution.**	X	X			
Only applies to some diagnostic products	Includes repair, rental and maintenance					
	**COICOP code 06.1.3 Assistive products for vision, hearing, mobility and daily living.**					

For peer review only

	**A. Purchase, repair, rental/maintenance of** (glasses for vision; hearing aids; crutches & wheelchairs; therapeutic footwear; walkers; pressure relief mattresses) **and all other assistive health products** .				X	X
**Type of information**	***Version-2***<br>**Level of *D*isaggregation – 44 Health items**	**Recall periods: D- days/ M-Months**				
		**15d**	**30d**	**3M**	**6M**	**12M**
	**COICOP code 06.2.3 Inpatient care services**					
**To be asked in bold**<br>Examples are given in brackets for more see explanatory notes<br>**If helpful can add at the end of the explanations: for patients with disabilities, the elderly (or those who requires permanent surveillance or constant help due to limited functional capacity)**	**I.LONG. Medical treatment and / or care that required overnight stay in a nursing home; (medical convalescent homes; palliative care establishments) or any other long term care medical facility**<br>**If yes**					
	**I.LONG.SP.1 medical services during the overnight long term care**				X	X
	**I.LONG.SP.2 medicines during the overnight long term care**				X	X
	**I.LONG.SP.3 medical products during the overnight long term care**				X	X
	**I.LONG.DT. diagnostic and laboratory tests**				X	X
	**I.LONG.NM. Non-medical cost for the patient (cooking, cleaning, accommodation) during the overnight long term care**				X	X
	**I.LONG.ER.1 Emergency transportation and rescue services by ambulance or other vehicles specially adjusted for medical purposes**				X	X
	**I.LONG.ER.2 Emergency transportation services and rescue by ordinary vehicles or airplanes (not specially adjusted for a medical purpose)**				X	X
	**I.CURR Medical and dental treatment that required an overnight stay in any type of facility** (e.g. hospitals, clinics) **excluding long term care medical facility**<br>**If yes**					
	**I.CURR.SP.1. medical, dental services during overnight stay**				X	X
	**I.CURR.SP.2. medicines for medical or dental treatment during overnight stay**				X	X
	**I.CURR.SP.3. medical products for medical or dental treatment during overnight stay**				X	X
	**I.CURR.DT. diagnostic and laboratory tests for medical or dental treatment during overnight stay (e.g. x-rays, scans, blood tests)**				X	X
	**I.CURR.NM. Non-medical costs for the patient (cooking, cleaning, accommodation)**				X	X
	**I.CURR.ER.1. emergency transportation and rescue services by ambulance or other vehicles specially adjusted for medical purposes**				X	X
	**I.CURR.ER.2. Emergency transportation services and rescue by ordinary vehicles or airplanes (not specially adjusted for a medical purpose)**				X	X
**Inclusion/Exclusion criteria to be specified when asking about the amount**<br>Applies to both I.LONG.SP.1 & I.CURR.SP.1	*If possible* exclude diagnostic and laboratory tests during the overnight stay I.LONG.DT/ I.CURR.DT. and emergency transportation services and emergency rescue (I.LONG.ER / I.CURR.ER)					
Applies to all I.LONG. & I.CURR	Excludes: non-emergency transportation and non-medical costs for patient's relative.					
	**COICOP code 06.2.1 Preventive care services**					
	**P.IMMV.MC. Immunization/vaccination services for maternal and child care**			X	X	
	**P.IMMV.OV. Travel and tourism vaccination, any other compulsory or voluntary immunization/vaccination service.**			X	X	
**Inclusion/Exclusion criteria to be specified when asking about the amount**<br>Applies to P.IMMV.OV	Includes; travel and tourism vaccination as well as any other immunization/vaccination service.					
Applies to both P.IMMV.MC and P.IMMV.OV	Excludes payments for the vaccine itself when separately invoiced from the service.					
	**P.OTHR. Other preventive services than immunization/vaccination**					
	**P.OTHR.GH.1 Family planning, counselling, prenatal/postnatal care services for both the mother and new born (during the six weeks or 42 days)**			X	X	
	**P.OTHR.GH.2 Child growth and development consultation visits and any other consultations to monitor "good" health of children and adults**			X	X	
	**P.OTHR.DI. screening, tests, consultations to detect communicable or non-communicable diseases before symptoms appear** (e.g. diabetes, heart problems)			X	X	
**Inclusion/Exclusion criteria to be specified when asking about the amount**<br>Applies to all P.IMMV & P.OTHR	Includes diagnostic and laboratory tests needed to provide preventive services					
	**COICOP code 06.4.2 Emergency transportation and emergency rescue**					
**Alternative wording: transportation for medical emergency reasons (e.g. by ambulance)**	**O.EMER. Patient emergency transportation services and emergency rescue services (excluding those associated with an overnight stay)**					

For peer review only

	**O.EMER.AV. by ambulance or other vehicles specially adjusted for medical purpose**	X	X			
	**O.EMER.NA. by ordinary vehicles or airplanes (not specially adjusted for a medical purpose)**	X	X			
**Inclusion/Exclusion criteria to be specified when asking about the amount**	Excludes non-emergency transportation services					
	**COICOP code 06.2.2 & 06.2.3 Outpatient dental & other outpatient services**					
	**O.DENT Outpatient dental care**					
	**In a hospital setting**					
	**O.DENT.CS.1 Dental consultations and services that did not require an overnight stay in a hospital setting**	X	X			
	**O.DENT.DT.1. Diagnostic and laboratory tests needed to provide dental consultations and services in a hospital setting**	X	X			
	**Outpatient settings (e.g. private practice, office, medical center, clinics, polyclinics)**					
	**O.DENT.CS.2. Dental consultations and services that did not require an overnight stay Outpatient settings (e.g. private practice, office, medical center, clinics, polyclinics)**	X	X			
	**O.DENT.DT.2. Diagnostic and laboratory tests needed to provide dental consultations and services Outpatient settings (e.g. private practice, office, medical center, clinics, polyclinics)**	X	X			
**Inclusion/Exclusion criteria to be specified when asking about the amount** Applies to O.DENT.CS..1 & O.DENT.CS.2	Excludes dental diagnostics services and laboratory tests needed to provide outpatient dental services (e.g. X-rays, blood tests)					
	**O.CRRL. Other medical consultations and services than dental and preventive that did not require an overnight stay**					
	**In a hospital setting**					
	**O.CRRL.CS.1.1 consultations and services of specialists (paediatricians, surgeons, cardiologists, ophthalmologist, mental health)**	X	X			
	**O.CRRL.CS.1.2 consultation and services of general doctors**	X	X			
	**O.CRRL.CS.1.3 consultation and services of nurses, midwifes and other health care practitioner**	X	X			
	**O.CRRL.DT.1 diagnostic and laboratory tests needed to provide other medical services that did not require an overnight stay in a hospital setting**	X	X			
	**Outpatient settings (e.g. private practice, office, medical center, clinics, polyclinics)**					
	**O.CRRL.CS.2.1 consultations and services of specialists (paediatricians, surgeons, cardiologists, ophthalmologist, mental health)**	X	X			
	**O.CRRL.CS.2.2 consultation and services of general doctors**	X	X			
	**O.CRRL.CS.2.3 consultation and services of nurses, midwifes and other health care practitioner**	X	X			
	**O.CRRL.DT.2 diagnostic and laboratory tests needed to provide other medical services that did not require an overnight stay outside a hospital setting**	X	X			
**Inclusion/Exclusion criteria to be specified when asking about the amount** Applies to all O.CRRL.CS.1 & O.CRRL.CS.2	excludes diagnostic and laboratory test needed to provide outpatient medical services (e.g. X-rays, blood/urine tests)					
	**COICOP code 06.1.1**					
	**M.HERH. Herbal medicines (tablets or syrups) and homeopathic products for consumption outside a health facility or institution.**	X	X			
	**M.MVCP. Medicines** (branded, generic)**, vaccines, oral contraceptives, vitamins and minerals for consumption outside a health facility or institution.**					
	**M.MVCP.IA. antibiotics**	X	X			
	**M.MVCP.IO.Other medicines (branded, generic, homeopathic) to treat (presumed or diagnosed) bacterial infections (e.g. malaria, diarrhoeas, dysentery, increased frequency of stools with or without blood and mucus in stools; worms infestations)**	X	X			
	**M.MVCP.CD. medicines to treat (presumed or diagnosed) non-communicable diseases or chronic diseases** (e.g. diabetes, hypertension)	X	X			
	**M.MVCP.FP. oral contraceptives and contraceptives in the form of injections**	X	X			
	**M.MVCP.SY medicines to treat fevers, pain and other symptoms (e.g. nausea; vomiting, constipation; inflammation)**	X	X			
	**M.MVCP.VM. vitamins, mineral**	X	X			
	**M.MVCP.OM. other medicines and pharmaceutical preparations not elsewhere specified**	X	X			
	**COICOP code 06.1. 2 medical diagnostic products, prevention and protective devices**					

For peer review only

	**D.** (Pregnancy tests, incontinence products and absorbent including diapers for the aging population, inhalers, mechanical contraceptives; insecticide treated mosquito nets, blood pressure devices) **and other medical health products for personal use outside a health facility or institution**.	X	X			
Inclusion/Exclusion criteria to be specified when asking about the amount<br>Only applies to some diagnostic products	Includes repair, rental and maintenance					
	**COICOP code 06.1.3 Assistive products for vision, hearing, mobility and daily living.**					
	**A.PURC. Purchase of** (glasses for vision; hearing aids; crutches & wheelchairs; therapeutic footwear; walkers; pressure relief mattresses) **and all other assistive health products** .				X	X
	**A.RRMN. Repair, rental/maintenance of** (glasses for vision; hearing aids; crutches & wheelchairs; therapeutic footwear; walkers; pressure relief mattresses) **and all other assistive health products** .				X	X

For peer review only

Type of information	*Version-3* Level of Disaggregation – 56 Health items	Recall periods: D- days/ M-Months				
		15d	30d	3M	6M	12M
	<u>**COICOP code 06.2.3 Inpatient care services**</u>					
<u>**To be asked in bold**</u><br>Examples are given in brackets for more see explanatory notes<br>**If helpful can add at the end of the explanations: for patients with disabilities, the elderly (or those who requires permanent surveillance or constant help due to limited functional capacity)**	**I.LONG. Medical treatment and / or care that required overnight stay in a nursing home; (medical convalescent homes; palliative care establishments) or any other long term care medical facility**<br>**If yes**					
	**I.LONG.SP.1 medical services during the overnight long term care**				X	X
	**I.LONG.SP.2 medicines during the overnight long term care**				X	X
	**I.LONG.SP.3 medical products during the overnight long term care**				X	X
	**I.LONG.DT. diagnostic and laboratory tests**				X	X
	**I.LONG.NM. Non-medical cost for the patient (cooking, cleaning, accommodation) during the overnight long term care**				X	X
	**I.LONG.ER.1 Emergency transportation and rescue services by ambulance or other vehicles specially adjusted for medical purposes**				X	X
	**I.LONG.ER.2 Emergency transportation services and rescue by ordinary vehicles or airplanes (not specially adjusted for a medical purpose)**				X	X
	**Medical and dental treatment that required an overnight stay in any type of facility** (e.g. hospitals, clinics) **excluding long term care medical facility**<br>**If yes**					
	**I.CURR.SP.1. medical, dental services during overnight stay**				X	X
	**I.CURR.SP.2 medicines for medical or dental treatment during overnight stay**				X	X
	**I.CURR.SP.3 medical products for medical or dental treatment during overnight stay**				X	X
	**I.CURR.DT. diagnostic and laboratory tests for medical or dental treatment during overnight stay (e.g. x-rays, scans, blood tests)**				X	X
	**I.CURR.NM. Non-medical costs for the patient (cooking, cleaning, accommodation)**				X	X
	**I.CURR.ER.1.emergency transportation and rescue services by ambulance or other vehicles specially adjusted for medical purposes**				X	X
	**I.CURR.ER.2. Emergency transportation services and rescue by ordinary vehicles or airplanes (not specially adjusted for a medical purpose)**				X	X
<u>**Inclusion/Exclusion criteria to be specified when asking about the amount**</u><br>Applies to both I.LONG.SP.1 & I.CURR.SP.1	*If possible* exclude diagnostic and laboratory tests during the overnight stay I.LONG.DT/ I.CURR.DT. and emergency transportation services and emergency rescue (I.LONG.ER / I.CURR.ER)					
Applies to all I.LONG. & I.CURR	Excludes: non-emergency transportation and non-medical costs for patient's relative.					
	<u>**COICOP code 06.2.1 Preventive care services**</u>					
	**P.IMMV.MC Immunization/vaccination services for maternal and child care**			X	X	
	**P.IMMV.OV Travel and tourism vaccination, any other compulsory or voluntary immunization/vaccination service.**			X	X	
<u>**Inclusion/Exclusion criteria to be specified when asking about the amount**</u><br>Applies to P.IMMV.OV	Includes; travel and tourism vaccination as well as any other immunization/vaccination service.					
Applies to both P.IMMV.MC and P.IMMV.OV	Excludes payments for the vaccine itself when separately invoiced from the service.					
	**P.OTHR. Other preventive services than immunization/vaccination**					
	**P.OTHR.GH.1 Family planning, counselling, prenatal/postnatal care services for both the mother and new born (during the six weeks or 42 days)**			X	X	
	**P.OTHR.GH.2 Child growth and development consultation visits and any other consultations to monitor "good" health**			X	X	
	**P.OTHR.DI. screening, tests, <u>consultations</u> to detect communicable or non-communicable diseases before symptoms appear** (e.g. diabetes, heart problems)			X	X	
<u>**Inclusion/Exclusion criteria to be specified when asking about the amount**</u><br>Applies to all P.IMMV & P.OTHR	Includes diagnostic and laboratory tests needed to provide preventive services					
	<u>**COICOP code 06.4.2 Emergency transportation and emergency rescue**</u>					
**Alternative wording: transportation for medical emergency reasons (e.g. by ambulance)**	**O.EMER. Patient emergency transportation services and emergency rescue services (excluding those associated with an overnight stay)**					

For peer review only

	**O.EMER.AV by ambulance or other vehicles specially adjusted for medical purpose**	X	X			
	**O.EMER.NA by ordinary vehicles or airplanes (not specially adjusted for a medical purpose)**	X	X			
**Inclusion/Exclusion criteria to be specified when asking about the amount**	Excludes non-emergency transportation services					
	**COICOP code 06.2.2 & 06.2.3 Outpatient dental & other outpatient services**					
	**O.DENT Outpatient dental care**					
	**O.DENT.CS.1 Dental consultations and services that did not require an overnight stay in a hospital setting**	X	X			
	**O.DENT.DT.1 Diagnostic and laboratory tests needed to provide dental consultations and services in a hospital setting**	X	X			
	**O.DENT.CS.2 Dental consultations and services that did not require an overnight stay Outpatient settings (e.g. private practice, office, medical center, clinics, polyclinics)**	X	X			
	**O.DENT.DT.2 Diagnostic and laboratory tests needed to provide dental consultations and services Outpatient settings (e.g. private practice, office, medical center, clinics, polyclinics)**	X	X			
**Inclusion/Exclusion criteria to be specified when asking about the amount** Applies to O.DENT.CS.1 & O.DENT.CS.2	Excludes dental diagnostics services and laboratory tests needed to provide outpatient dental services (e.g. X-rays, blood tests)					
	**O.CRRL. Other medical consultations and services than dental and preventive that did not require an overnight stay**					
	**In a hospital setting**					
	**O.CRRL.CS.1.1 consultations and services of specialists (paediatricians, surgeons, cardiologists, ophthalmologist, mental health)**	X	X			
	**O.CRRL.CS.1.2 consultation and services of general doctors**	X	X			
	**O.CRRL.CS.1.3 consultation and services of nurses, midwifes and other health care practitioner**	X	X			
	**O.CRRL.DT.1 diagnostic and laboratory tests needed to provide other medical services that did not require an overnight stay in a hospital setting**	X	X			
	**Outpatient settings (e.g. private practice, office, medical center, clinics, polyclinics)**					
	**O.CRRL.CS.2.1 consultations and services of specialists (paediatricians, surgeons, cardiologists, ophthalmologist, mental health)**	X	X			
	**O.CRRL.CS.2.2 consultation and services of general doctors**	X	X			
	**O.CRRL.CS.2.3 consultation and services of nurses, midwifes and other health care practitioner**	X	X			
	**O.CRRL.DT.2. diagnostic and laboratory tests needed to provide other medical services that did not require an overnight stay outside a hospital setting**	X	X			
**Inclusion/Exclusion criteria to be specified when asking about the amount** Applies to all O.CRRL..CS1 & O.CRRL..CS.2	excludes diagnostic and laboratory test needed to provide outpatient medical services (e.g. X-rays, blood/urine tests)					
	**COICOP code 06.1.1**					
	**M.HERH. Herbal medicines (tablets or syrups) and homeopathic products for consumption outside a health facility or institution.**					
	**M.HERH.PR prescribed**	X	X			
	**M.HERH.OC over-the-counter (self-prescription)**	X	X			
	**M.MVCP. Medicines** (branded, generic)**, vaccines, oral contraceptives, vitamins and minerals for consumption outside a health facility or institution.**					
	**prescribed**					
	**M.MVCP.PR.IA. antibiotics**	X	X			
	**M.MVCP.PR.IO. Other medicines (branded, generic, homeopathic) to treat (presumed or diagnosed) bacterial infections (e.g. malaria, diarrhoeas, dysentery, increased frequency of stools with or without blood and mucus in stools; worms infestations)**	X	X			
	**M.MVCP.PR.CD. medicines to treat (presumed or diagnosed) non-communicable diseases or chronic diseases** (e.g. diabetes, hypertension)	X	X			
	**M.MVCP.PR.FP. oral contraceptives and contraceptives in the form of injections**	X	X			
	**M.MVCP.PR.SY. medicines to treat fevers, pain and other symptoms (e.g. nausea; vomiting, constipation; inflammation)**	X	X			
	**M.MVCP.PR.VM. vitamins, mineral**	X	X			
	**M.MVCP.PR.OM. other prescribed medicines and pharmaceutical preparations not elsewhere specified**	X	X			

For peer review only

	over-the-counter (self-prescription)					
	**M.MVCP.OC.IA. antibiotics**	X	X			
	**M.MVCP.OC.IO Other medicines (branded, generic, homeopathic) to treat bacterial infections (e.g. malaria, diarrhoeas, dysentery, increased frequency of stools with or without blood and mucus in stools; worms infestations)**	X	X			
Examples on NCD should list the most prevalent in country/site	**M.MVCP.OC.CD medicines to treat (presumed or diagnosed) non-communicable diseases or chronic diseases** (e.g. diabetes, hypertension)	X	X			
	**M.MVCP.OC.FP oral contraceptives and contraceptives in the form of injections**	X	X			
	**M.MVCP.OC.SY medicines to treat fevers, pain and other symptoms (e.g. nausea; vomiting, constipation; inflammation)**	X	X			
	**M.MVCP.OC.VM vitamins, mineral**	X	X			
	**M.MVCP.OC.OM other self-prescribed medicines and pharmaceutical preparations not elsewhere specified**	X	X			
	COICOP code 06.1. 2 medical diagnostic products, prevention and protective devices for personal use outside a health facility or institution					
	**D.DIAG.** (pregnancy tests; thermometers, glucose-meters, blood pressure meters) **and other medical diagnostic products**	X	X			
	**D. PREP** condoms and other mechanical contraceptive devices, masks , medicinal stockings (e.g. compression stockings), medicinal gloves, insecticide treated mosquito – nets **and other prevention, protective medical devices**	X	X			
	**D.TRTM** inhalers, syringes, humidifiers, nebulizers, hot bags, ice packs, first aid kits, bandages **and other treatment devices for personal use**	X	X			
Inclusion/Exclusion criteria to be specified when asking about the amount<br>Only applies to some diagnostic products	Includes repair, rental and maintenance					
	COICOP code 06.1.3 Assistive products for vision, hearing, mobility and daily living.					
	**A.PURC.VH. Purchase of** glasses for vision; white canes, glass eyes, contact lenses, hearing aids and **other assistive products for vision and hearing**				X	X
	**A.RRMN.VH. Repair, rental/maintenance of assistive health products for vision and hearing**				X	X
	**A.PURC.MD. Purchase of** crutches & wheelchairs; therapeutic footwear; walkers; pressure relief mattresses **and all other assistive health products for mobility and daily living.**				X	X
	**A.RRMN.MD. Repair, rental/maintenance of assistive health products for mobility and daily living.**				X	X

For peer review only

Supplementary material 3: Supplementary tables

Table 1: Demographic Characteristics of households for matched versus unmatched households and households with versus households without OOPs

	Households with any expenditure		Households without any expenditure		Matched Households		Unmatched Households	
Total number of households	N=2093		N=914		N=1300		N=1698	
	n	%	n	%	n	%	n	%
**Sex**								
Male	1332	64	589	64	841	65	1074	63
**Marital status**								
Married	1326	63	519	57	827	64	1013	60
**Level of Education**								
No education	1116	53	474	52	656	50	931	55
Primary	420	20	196	21	262	20	349	21
Junior high school	292	14	128	14	195	15	223	13
Senior high school	97	5	43	5	71	5	70	4
Vocational/Technical/College/Graduate	167	8	73	8	116	9	125	7
**Religion**								
Christians	1114	53	460	50	730	56	841	50
Islam	162	8	81	9	107	8	136	8
Traditional	708	34	326	34	387	30	638	38
No religion	110	5	47	5	76	6	83	5
**Age group**								
15 - 19	96	5	37	4	60	5	74	4
20-34	138	7	58	6	91	7	102	6
35 - 64	1228	59	543	59	781	60	989	58
65 +	630	30	276	30	368	28	533	31
Mean age (SD)	55	17	55	17	54	17	55	17
**Household size**								
1 person	108	**5**	103	**11**	70	**5**	141	**8**
2-5 persons	1010	**48**	516	**56**	616	**47**	906	**53**
6 and above	975	**47**	295	**32**	614	**47**	652	**38**

Table 2: Summary of mean OOPs for unmatched and matched households by spending categories

	Questionnaire Version 1				Questionnaire Version 2				Questionnaire Version-3			
	11 disaggregated health items				44 disaggregated health items				56 disaggregated health items			
		Unmatched Household OOPs		Matched Household OOPs		Unmatched Household OOPs		Matched Household OOPs		Unmatched Household OOPs		Matched Household OOPs
	N	Mean (SD)	N	Mean (SD)	N	Mean (SD)	N	Mean (SD)	N	Mean(SD)	N	Mean (SD)
Outpatient	81	64 (135)	44	66 (128)	181	43 (130)	126	43 (150)	105	44 (78)	47	31 (78)
Inpatient	171	319 (527)	91	226 (279)	177	398 (809)	99	314 (504)	193	287 (716)	100	215 (456)
Medicines	487	41 (140)	302	36 (157)	560	29 (78)	381	25 (56)	609	29 (76)	354	30 (90)
Preventive care	137	59 (95)	80	49 (83)	92	34 (53)	67	31 (53)	46	31 (44)	22	20 (29)
Other medical services	8	203 (201)	2	43 (4)	5	113 (217)	3	15 (10.5)	2	12 (4)	2	12 (4)
Health products	36	71 (133)	19	84 (176)	25	160 (250)	14	178 (289)	18	165 (232)	7	6 (5)

Table 3: Summary of mean OOPs for matched households and provider data by spending categories

	Questionnaire Version 1				Questionnaire Version 2				Questionnaire Version-3			
	11 disaggregated health items				44 disaggregated health items				56 disaggregated health items			
		Provider OOPs	Household OOPs	Estimated ratio (HH/provider) of the means (95% CI)		Provider OOPs	Household OOPs	Estimated ratio (HH/provider) of the means (95%CI)		Provider OOPs	Household OOPs	Estimated ratio (HH/provider) of the means (95%CI)
	N	Mean (SD)	Mean (SD)		N	Mean(SD)	Mean (SD)		N	Mean(SD)	Mean (SD)	
Outpatient	44	34 (46)	66 (128)	1.92	126	22 (52)	43 (150)	1.98	47	14 (15)	31 (78)	2.23
Inpatient	91	49 (74)	226 (279)	6.5	99	48 (97)	314 (504)	6.54	100	21 (38)	215 (456)	10.34
Medicines	302	26 (67)	36 (157)	1.39	381	21 (57)	25 (56)	1.19	354	12 (21)	30 (90)	2.54
Preventive care	80	32 (47)	49 (83)	1.53	67	38 (75)	31 (53)	0.81	22	12 (18)	20 (29)	1.73
Other medical services	2	11 (11)	43 (4)	4.05	3	16 (7)	15 (10.5)	0.94	2	9 (9)	12 (4)	1.33
Health products	19	44 (61)	84 (176)	1.9	14	21 (28)	178 (289)	8.66	7	36 (31)	6 (5)	0.17

For peer review only

STROBE Statement—checklist of items that should be included in reports of observational studies

	Item No	Recommendation	Page No
**Title and abstract**	1	(*a*) Indicate the study's design with a commonly used term in the title or the abstract	1
		(*b*) Provide in the abstract an informative and balanced summary of what was done and what was found	2
**Introduction**			
Background/rationale	2	Explain the scientific background and rationale for the investigation being reported	4
Objectives	3	State specific objectives, including any prespecified hypotheses	4
**Methods**			
Study design	4	Present key elements of study design early in the paper	6
Setting	5	Describe the setting, locations, and relevant dates, including periods of recruitment, exposure, follow-up, and data collection	5
Participants	6	(*a*) *Cohort study*—Give the eligibility criteria, and the sources and methods of selection of participants. Describe methods of follow-up<br>*Case-control study*—Give the eligibility criteria, and the sources and methods of case ascertainment and control selection. Give the rationale for the choice of cases and controls<br>*Cross-sectional study*—Give the eligibility criteria, and the sources and methods of selection of participants	6
		(*b*) *Cohort study*—For matched studies, give matching criteria and number of exposed and unexposed<br>*Case-control study*—For matched studies, give matching criteria and the number of controls per case	
Variables	7	Clearly define all outcomes, exposures, predictors, potential confounders, and effect modifiers. Give diagnostic criteria, if applicable	7,8
Data sources/ measurement	8*	For each variable of interest, give sources of data and details of methods of assessment (measurement). Describe comparability of assessment methods if there is more than one group	6
Bias	9	Describe any efforts to address potential sources of bias	7,8
Study size	10	Explain how the study size was arrived at	6
Quantitative variables	11	Explain how quantitative variables were handled in the analyses. If applicable, describe which groupings were chosen and why	
Statistical methods	12	(*a*) Describe all statistical methods, including those used to control for confounding	7,8
		(*b*) Describe any methods used to examine subgroups and interactions	
		(*c*) Explain how missing data were addressed	
		(*d*) *Cohort study*—If applicable, explain how loss to follow-up was addressed<br>*Case-control study*—If applicable, explain how matching of cases and controls was addressed<br>*Cross-sectional study*—If applicable, describe analytical methods taking account of sampling strategy	7,8
		(*e*) Describe any sensitivity analyses	

Continued on next page

For peer review only - http://bmjopen.bmj.com/site/about/guidelines.xhtml

**Results**			
Participants	13*	(a) Report numbers of individuals at each stage of study—eg numbers potentially eligible, examined for eligibility, confirmed eligible, included in the study, completing follow-up, and analysed	9
		(b) Give reasons for non-participation at each stage	
		(c) Consider use of a flow diagram	9
Descriptive data	14*	(a) Give characteristics of study participants (eg demographic, clinical, social) and information on exposures and potential confounders	10
		(b) Indicate number of participants with missing data for each variable of interest	
		(c) *Cohort study*—Summarise follow-up time (eg, average and total amount)	
Outcome data	15*	*Cohort study*—Report numbers of outcome events or summary measures over time	
		Case-control study—Report numbers in each exposure category, or summary measures of exposure	
		Cross-sectional study—Report numbers of outcome events or summary measures	11
Main results	16	(*a*) Give unadjusted estimates and, if applicable, confounder-adjusted estimates and their precision (eg, 95% confidence interval). Make clear which confounders were adjusted for and why they were included	13
		(*b*) Report category boundaries when continuous variables were categorized	12,13
		(*c*) If relevant, consider translating estimates of relative risk into absolute risk for a meaningful time period	
Other analyses	17	Report other analyses done—eg analyses of subgroups and interactions, and sensitivity analyses	
**Discussion**			
Key results	18	Summarise key results with reference to study objectives	14
Limitations	19	Discuss limitations of the study, taking into account sources of potential bias or imprecision. Discuss both direction and magnitude of any potential bias	15
Interpretation	20	Give a cautious overall interpretation of results considering objectives, limitations, multiplicity of analyses, results from similar studies, and other relevant evidence	14,15
Generalisability	21	Discuss the generalisability (external validity) of the study results	15
**Other information**			
Funding	22	Give the source of funding and the role of the funders for the present study and, if applicable, for the original study on which the present article is based	16

*Give information separately for cases and controls in case-control studies and, if applicable, for exposed and unexposed groups in cohort and cross-sectional studies.

For peer review only - http://bmjopen.bmj.com/site/about/guidelines.xhtml

BMJ Open

Effect of specificity of health expenditure questions in the measurement of out-of-pocket health expenditure: Evidence from field experimental study in Ghana

Journal:	*BMJ Open*
Manuscript ID	bmjopen-2020-042562.R1
Article Type:	Original research
Date Submitted by the Author:	04-Mar-2021

Complete List of Authors:	Agorinya, Isaiah; University of Health and Allied Sciences, Epidemiology and Biostatistics; Swiss Tropical and Public Health Institute, Epidemiology and Public Health<br>Ross, Amanda; Swiss Tropical and Public Health Institute<br>Flores, Gabriela; World Health Organization<br>TanTorres Edejer, Tessa; World Health Organization, Global Programme on evidence for Health Polic<br>Dalaba, Maxwell Ayindenaba; Navrongo Health Research Centre,<br>Mensah, Nathan; University of Cape Coast, Department of Health Information Management<br>Le My, Lan; Swiss Tropical and Public Health Institute, Epidemiology and Public Health; University of Basel<br>Dessie, Yadeta; Haramaya University, Public Health<br>Sumboh, Jemima; Navrongo Health Research Centre<br>Oduro, Abraham; Navrongo Health Research Centre, Epidemiology<br>Akazili, J; Ghana Health Service<br>Tediosi, Fabrizio; Schweizerisches Tropen- und Public Health-Institut

**Primary Subject Heading**:	Health economics
Secondary Subject Heading:	Health economics, Health policy, Health services research
Keywords:	HEALTH ECONOMICS, EPIDEMIOLOGY, STATISTICS & RESEARCH METHODS

SCHOLARONE™
Manuscripts

I, the Submitting Author has the right to grant and does grant on behalf of all authors of the Work (as defined in the below author licence), an exclusive licence and/or a non-exclusive licence for contributions from authors who are: i) UK Crown employees; ii) where BMJ has agreed a CC-BY licence shall apply, and/or iii) in accordance with the terms applicable for US Federal Government officers or employees acting as part of their official duties; on a worldwide, perpetual, irrevocable, royalty-free basis to BMJ Publishing Group Ltd ("BMJ") its licensees and where the relevant Journal is co-owned by BMJ to the co-owners of the Journal, to publish the Work in this journal and any other BMJ products and to exploit all rights, as set out in our licence.

The Submitting Author accepts and understands that any supply made under these terms is made by BMJ to the Submitting Author unless you are acting as an employee on behalf of your employer or a postgraduate student of an affiliated institution which is paying any applicable article publishing charge ("APC") for Open Access articles. Where the Submitting Author wishes to make the Work available on an Open Access basis (and intends to pay the relevant APC), the terms of reuse of such Open Access shall be governed by a Creative Commons licence – details of these licences and which Creative Commons licence will apply to this Work are set out in our licence referred to above.

Other than as permitted in any relevant BMJ Author's Self Archiving Policies, I confirm this Work has not been accepted for publication elsewhere, is not being considered for publication elsewhere and does not duplicate material already published. I confirm all authors consent to publication of this Work and authorise the granting of this licence.

For peer review only

Effect of specificity of health expenditure questions in the measurement of out-of-pocket health expenditure: Evidence from field experimental study in Ghana

Isaiah Awintuen Agorinya*[1, 2,4,5], Amanda Ross[1,4], Gabriela Flores[6], Tessa Tan-torres Edejer[6], Maxwell Dalaba[3,5], Nathan Kumasenu Mensah[7], Le My Lan[1,4,8], Yadeta Dessie Bacha[9], Jemima Sumboh[3], Abraham Oduro[3], James Akazili[2,3], Fabrizio Tediosi[1,4]

[1]Swiss Tropical and Public Health Institute,
[2]INDEPTH-Network Secretariat, Accra, Ghana
[3]Navrongo Health Research Centre, Navrongo, Ghana
[4]University of Basel, Basel, Switzerland
[5]University of Health and Allied Sciences, Ho, Ghana
[6] World Health Organization (WHO), Geneva, Switzerland
[7]Department of Health Information Management, University of Cape Coast, Ghana
[8]FilaBavi Health and Demographic Surveillance Site, Hanoi, Vietnam
[9]Department of Public Health, College of Health and Medical Sciences, Haramaya University, Harar, Ethiopia

[*] **Corresponding author**

Email: iagorinya@gmail.com (IAA)

For peer review only - http://bmjopen.bmj.com/site/about/guidelines.xhtml

Abstract

Background: The effect of number of health items on OOPs has been identified as a source of bias in measuring OOPs. Evidence comes mostly from cross-sectional comparison of different survey instruments to collect data on OOPs. Very few studies have attempted to validate these questionnaires, or distinguish bias arising from the comprehensiveness of the OOPs list versus specificity of OOPs questions.

Objectives: This study aims to estimate biases arising from the specificity of OOPs questions by comparing provider and household's information.

Methods: A generic questionnaire to collect data on household's OOPs was developed following the nomenclature proposed in division 6 of the COICOP2018. The four categories within such division are used to set the comprehensiveness of the OOPs list, the specificity within each category was tailored to the design of the nationally representative living standard survey in Ghana where a field experiment was conducted to test the validity of different versions. Households were randomized to 11, 44 or 56 health items. Using data from provider records as the gold standard, we compared the mean positive OOPs, and estimated the mean ratio and variability in the ratio of household expenditures to provider data for the individual households using the Bland-Altman method of assessing agreement.

Findings: We found evidence of a difference in the overall mean ratio in the specificity for OOPs in inpatient care and medications. Within each of these two categories, a more detailed disaggregation yielded lower OOPs estimates than less detailed ones. The level of agreement between household and provider OOPs also decreased with increasing specificity of health items.

Conclusion: Our findings suggest that, for inpatient care and medications, systematically decomposing OOPs categories into finer sub-classes tend to produce lower OOPs estimates. Less detailed items produced more accurate and reliable OOPs estimates in the context of a rural setting.

Keywords

Health expenditure, out-of-pocket payments, universal health coverage, household survey, specificity, validation, Ghana

For peer review only - http://bmjopen.bmj.com/site/about/guidelines.xhtml

Strengths and Limitations of this study

- The main strength of this study is the use of provider data as a ‘gold standard’ to validate self-reported household out-of-pocket health expenditures in a rural African setting.
- This study has quantified the level of agreement and variability in current household survey instruments that uses different levels of health expenditure items to estimate OOPs.
- The study validated spending categories contained in the latest COICOP revisions (COICOP 2018) which has previously not been done
- Generation of provider data posed as a major limitation to this study as this was not a routine practice by health providers.
- Sample sizes for each spending category was largely affected by the fraction of households reported OOPs that accurately matched with provider data

For peer review only

Introduction

Household out-of-pocket health payments (OOPs) are direct payments for services from households' primary income or savings with no third-party payer involved [1]. OOPs are an important measure of performance of the health financing system. They are used to monitor to what extent voluntary and unpredictable payments are used to mobilize money within the health system and the impact of such payments on the household's living standard and ability to spend on other basic needs [2–6]

An important source of information to track OOPs are household surveys, especially in countries where much private health care financing occurs without the generation of linked, reliable and comprehensive routine data. Household consumption and expenditure surveys, household utilization and expenditure surveys, and health surveys with information on health expenditures can be used to gather data on OOPs.

These surveys are not standardized as they can differ, among other things, in the comprehensiveness (the number of main categories covered by the survey) as well as the level of detail (the specificity within each main category of health product or service) [7–12]. Lu et al (2009) comparing estimates from 50 countries using World Health Survey (WHS) data reported that, –if there was one rather than eight health categories, then the average reported health spending tended to be lower. The number of questions varies: Heijink (2011) reviewed survey questionnaires for 114 countries and found that the number of health expenditure questions mostly vary between 1 and 25, with some outliers falling over this range [11]. Lavado et al (2013), also found the number of questions on health expenditure to range from 1 to 274 in 214 surveys and estimated that an additional question on OOPs increases health expenditure share by 1% [13]. However, neither Lavado et al, nor Heijink et al distinguished bias arising from comprehensiveness of the OOPs list versus specificity within each category.

To the best of our knowledge, no validation study has so far been conducted to assess the effect of the specificity of the expenditure questions on the accuracy and reliability of reported OOPs in the context of low-middle income countries . In this paper, we compare household responses on health expenditure questions to the corresponding health provider data in order to assess and quantify agreement. We use questionnaires with three different levels of detail to assess the effect of the specificity of health items on the accuracy of reported OOPs

For peer review only

For peer review only - http://bmjopen.bmj.com/site/about/guidelines.xhtml

Methods

Strategy

The overall project, in which this study is nested, assesses the impact of different survey characteristics, such as specificity, recall period, and whether the survey is door to door or telephone based, on the accuracy of household-reported out-of-pocket payments (iHOPE). It was implemented in three health and demographic surveillance sites (HDSS) of the INDEPTH-Network located in northern Ghana (Navrongo health and demographic surveillance site, mostly rural), Burkina Faso (Ouagadougou demographic surveillance site, informal urban setting) and Vietnam (FilaBavi demographic surveillance site, mixed rural/urban setting). The INDEPTH-Network platform provided the project with the opportunity to identify and track households and link them to health provider records to be able to validate household reported out-of-pocket expenditures. In this paper, we report the study on the effect of the specificity of health expenditure categories on reported OOPs conducted in Navrongo HDSS (Ghana). For this experiment, three versions of health expenditure modules with different level of specificity of health expenditure categories were developed and adapted to the structure of the Ghana living standards survey 6 (GLSS6) instrument [14]. These three versions were all comprehensive in that they all captured the major health care consumption groups that constitute the main categories of health care expenditures individuals are confronted with as identified by the classification of household final consumption (COICOP), 2018 version [15]. They differed in the level of detail (specificity) within each class. Health expenditures were first compared across versions without any gold standard. This is what most studies to date have been able to analyze. The value added of this study is the use of health provider records to identify the level of agreement between two different sources (provider records versus household reports) of the same health expenditures made by household members, and to compare the level of agreement between the different questionnaire versions. .

Study settings

This study was implemented at the Navrongo HDSS site located in the Northern region of Ghana. The site has two administrative districts with an estimated total population of 160,000. The site has one public hospital, one health research centre, one private clinic, seven health centers, and 27 community-based health compounds. A number of pharmacy shops, chemical and drug shops, petty traders and peddlers, herbalists, faith-based and traditional healers also operate in the area. The research centre collects vital socio-demographic data every four months while household characteristics and assets are collected every two years [16].

For peer review only - http://bmjopen.bmj.com/site/about/guidelines.xhtml

Study context: Ghana's National Health Insurance Scheme

Ghana enacted legislation (National Health Insurance Act 2003 (Act 650) and implemented a national health insurance scheme (NHIS) aimed at reducing out of pocket payments that were estimated to account for up to 48% of the total health expenditure [17]. The NHIS offers free access to a package of diagnostic, inpatient and outpatient services covering 95% of conditions afflicting Ghanaians with services including primary curative care to care at tertiary facilities but challenges within the operationalization of the scheme are thought to expose subscribers to OOPs [18]. The NHIS is intended to cover the whole population with formal workers automatically covered through deductible social contributions, informal workers registered through annual premium payments while vulnerable people are exempted from paying premiums.

The scheme is largely financed through tax and a small proportion from contributions and donations. In 2014, the scheme covered only about 40% of Ghana's population (10.5 million active subscribers) with about 69% of these exempted from any form of payment to the scheme [19]. The exempt groups include indigent people, pregnant women and very poor households covered by a social intervention programme called Livelihood Empowerment Against Poverty (LEAP). Household members who enroll into the Ghana NHIS are assured of free services within the scope of the NHIS beneficiary package. However, subscribers to the NHIS may be exposed to OOPs when accessing health providers for medicines, laboratory tests and other consumables which may not be available at the provider due for example to stock-outs [18] or non-accredited NHIS provider. The uninsured population are required to pay out-of-pocket to be able to access health care [19].

In Ghana OOPs are mostly incurred by households that are not registered with the NHIS, for services that are not included in the NHIS benefit package, or due to challenges within the operationalization of the health insurance scheme or households accessing health care from a non-health insurance accredited private health provider. Informal payments for healthcare are not common in the study area (this was investigated during piloting) as seen in other areas and therefore has are not included in this study.

Study design

This study uses health provider records as the 'gold standard' to compare to household reported OOPs, recognizing that the provider records are not a perfect gold standard in the absence of a pre-existing formal recording system in place. Two sets of data were collected in this study. The first set of data was captured from households in a cross-sectional field survey conducted between May 2017 and December 2017 and the second set of data was obtained from health provider records within the same period.

Households were randomized to one of three versions of a household questionnaire on consumption expenditure for face-to-face interview. All three versions of the questionnaire were fielded during the same time period and included questions on out-of-pocket health spending for inpatient care services, preventive

For peer review only - http://bmjopen.bmj.com/site/about/guidelines.xhtml

care services, other outpatient care services, other health services, medicines and health products. They however differed in the level of specificity within each one of these main OOPs categories. The versions were labeled; version-1 for the questionnaire with 11 items covering the 6 health categories previously mentioned version-2 and 3 for the questionnaire versions with a maximum of 44 and 56 items respectively (See Figure 1 and Table 1 in supplementary material 1). All three versions of the instrument used similar recall periods of 4 weeks for items listed under the categories medicines, other outpatient services, other health services; 12 months for inpatient care services and other health products; and 6 months for OOPs related to preventive care services. Supplementary material 1 discusses in detail how the structure of the questionnaire was obtained. The specificity of OOPs categories is also available from Supplementary material 2.

We created a database of provider records to validate the household reported OOPs. Both private and public health care providers within the study area were engaged by the project team to extract records covering a 13 month period which in some cases (mostly for private providers) also required improving the recording of provider records. Health expenditures reported by any member of the household were tracked and a corresponding health provider record obtained to create a matched sample for validation. A detailed description of the matching procedure can be seen in Supplementary material one under the section Matching. Matched sample in this study refers to one-on-one paired records households reported OOPs to heath provider recorded OOPs for the same reason of incurring the health cost. Unmatched households in this study also refers to all OOPs reported by households without pairing such expenditures to corresponding health provider records. Household heads were the most common respondents for the household survey but in some cases, other individuals within the household were nominated by the household head to provide responses.

Study population and Sampling

All households registered under the Navrongo demographic surveillance site database constituted our study population. The households were randomly selected. The sample size for the household cross-sectional survey was based on estimating the agreement between two quantitative measurements. We followed the Bland and Altman approach which suggests a rule of thumb that 100-200 observations is adequate for sufficient precision when assessing agreement [20]. Based on unpublished district health management report in the study area, the probability of spending on outpatient for a 2-week recall period was 15.5% and 10% for a 12-month recall for inpatient care. To achieve a minimum of 100 households with reported inpatient care (outpatient spending is included in this sample), the sample size required would be 1000 households plus 10% non-response rate which gives a total of 1100 households per questionnaire version, and 3300 households for all three versions.

For peer review only - http://bmjopen.bmj.com/site/about/guidelines.xhtml

Data analysis

We carried out separate analyses for the unmatched and matched data to assess the effect of the number of health items on the amount and accuracy of the reported OOPs. We assessed the consistency of the findings in these two analyses. For the unmatched samples, we summarized the OOPs by spending categories and household characteristics for each questionnaire version using arithmetic means and standard deviations for continuous variables and proportions for categorical variables. Summarizing and comparing household reported OOPs are consistent with the methodological approach used in most studies to date without information from the provider [11]. We summarized the effect of number of items on mean reported OOPs by spending category using the ratio of each category to the reference version (version-1 which had 11 items). The confidence intervals for the ratio were calculated using the bootstrapping method. Only four categories of spending were considered instead of six due to the small number of households reporting any OOPs for health products and other health services.

For the matched samples, we adopted the Bland-Altman approach [21,22] to assess the level of agreement between household reported OOPs and corresponding provider records. We first estimated the agreement for each questionnaire version separately. We calculated the ratio of individual household to matched provider OOPs. The ratio was preferred to the simple difference since it had a reasonably constant distribution over the range of the provider OOPs, whereas the difference between household and provider OOPs was dependent on the level of provider OOPs. The log-transformation of the ratio was used for analysis due to the skewed distribution of the ratios [21,22]. For each questionnaire version, we estimated the overall mean ratio using the geometric mean of the ratios and the variability using the 95% limits of agreement (LoA) (i.e. limits within which 95% of the ratios are expected to lie). To compare the three versions of the questionnaire and assess the effect of number of health items on OOPs, we compared the overall mean ratio and variability using regression models proposed by Bland-Altman [22]. Specifically, for the mean ratio, we fitted a regression model with the log ratio of household to provider OOPs as the outcome variable and questionnaire version as an explanatory variable. This provides an estimate of the effect of the questionnaire version on the bias, measured by the mean log ratio with corresponding confidence interval and p-value. We included a random effect parameter to account for the clustering of the households within clusters defined by the Navrongo HDSS. For the effect of the questionnaire version on the variability, a second regression model was used with the outcome of the absolute values of the residuals previously obtained against the questionnaire version. Since the dependent variable in the first regression is on the log scale, all results were transformed to the ratio scale for ease of interpretation. Questionnaire version 1 which has the least number of health items (11 items) was used as the reference group for the regression models. Data was analyzed using STATA Version 14, Stata Corp.

For peer review only - http://bmjopen.bmj.com/site/about/guidelines.xhtml

Ethics approval and consent to participate

The Ethical Review Board of the Navrongo Health Research Centre, Ghana (NHRCIRB217) approved for the conduct of the study. Informed consent was obtained from all study participants.

Patient and Public Involvement

Per the design and objective of this study, it was not appropriate to involve the public in the design, or conduct, or reporting, or dissemination plans of our research.

For peer review only

For peer review only - http://bmjopen.bmj.com/site/about/guidelines.xhtml

Results

Sample distribution and matching

A total of 3300 households were sampled and randomized to receive one of the three questionnaire versions. The response rate was 83% in version 1, 94% in version 2 and 94% in version 3. Approximately 70% of households reported any form of OOP across all three questionnaire versions as expected. In terms of the matching of household expenditures to provider data, the proportion of households that matched was 60% in version 1, 65% in version 2 and 55% in version 3. Figure 1 below shows a flow chart that summarizes the sample distribution, the proportion of households incurring OOPs and the overall proportion of OOPs that matched with provider records in each questionnaire version.

Insert figure 1 here

Socio-demographic characteristics of households

A total of 3023 households were interviewed out of 3300 sampled. Of these, 64% of the household heads were males, the majority (89%) of the household heads were above 34 years of age, 62% of the household heads were married, slightly more than half (53%) of household heads did not have any formal education or were Christians and the mean age was 55 years (17 SD). The majority of households had more than one member with 51% having 2-5, and 42% with 6 or more (Table 1). From Table S1 in supplementary material 3, the distribution of demographic characteristics was similar between households in the three questionnaire versions, households with health expenditure versus those without, and households with matched provider records versus households without successful matched provider records. (Supplementary material 3: Table S1).

For peer review only

Table 1: Demographic Characteristics of household and household head

	Questionnaire version 1		Questionnaire version 2		Questionnaire version 3		All Questionnaire versions combined	
Total number of households	N=925		N=1062		N=1036		N=3023	
	n	%	n	%	n	%	n	(%)
**Gender**								
Male	582	63	705	66	647	62	1934	64
**Marital status**								
Married	566	61	687	65	606	59	1859	62
**Level of Education**								
No education	451	49	574	54	572	55	1597	53
Primary	200	22	214	20	202	20	616	20
Junior high school	144	16	141	13	136	13	421	14
Senior high school	44	5	51	5	49	5	144	5
Vocational/Technical/College/Graduate	86	9	82	8	77	7	245	8
**Religion**								
Christians	519	56	501	47	571	55	1591	53
Islam	54	6	148	14	41	4	243	8

For peer review only - http://bmjopen.bmj.com/site/about/guidelines.xhtml

Traditional	314	34	355	33	362	35	1031	34
No religion	38	4	58	6	62	6	158	5
**Age group**								
15 – 19	40	4	56	5	43	4	139	5
20-34	51	6	77	7	67	7	195	6
35 – 64	572	62	621	59	589	57	1782	59
65 +	262	28	308	29	337	33	907	30
Mean age (SD)	55	17	54	17	56	17	55	17
**Household size**								
1 person	67	7	82	8	64	6	213	7
2-5 persons	418	45	549	52	566	55	1533	51
6 and above	441	48	432	41	403	39	1276	42

Proportion reporting any OOPs by spending category

Overall, 2969 households provided information about household health spending of which 71% of households reported any form of OOPs. The proportion of all households reporting OOPs for inpatient care over the 12 month recall period was 18%, for preventive care over the past 6 month recall period 9%, for outpatient care within the past 4 weeks 12%, and slightly more than half (56%) of the households also reported having incurred OOPs on medications over the past 4 weeks regardless of the number of health items (Table 2).

Table 2: Health expenditures – Proportion of households reporting positive OOPs by spending category

	Questionnaire version-1 N=901			Questionnaire version -2 N=1032			Questionnaire version -3 N=1036		
number of households with positive health by category: new COICOP classification	Number of health items	n	%	Number of health items	n	%	Number of health items	N	%
inpatient care services	2	170	19	14	177	17	14	193	19
preventive services	2	137	15	5	92	9	5	46	4
Other health services	1	9	1	2	5	0.5	2	2	0.2
Outpatient	2	81	9	12	181	18	12	105	10
Medicines	2	487	54	9	560	54	16	609	59
health products	2	36	4	2	25	2	7	18	2
number of households with any health expenditure	11	645	71	44	720	70	56	736	71

Mean OOPs reported by households by spending category (unmatched analysis)

The mean household OOPs tended to be larger for lower numbers of health expenditure items (less specificity) in all main spending categories except health products where there are few households with spending, and inpatient care where there was no consistent pattern with increasing specificity. The differences in specificity between questionnaire versions were significant for only preventive care and medicines (Table 3). Table S2 and Table S3 in Supplementary material 3 also shows the summary of mean OOPs compared for matched and unmatched households versus provider estimates across the three versions and by spending categories. The tables show that, the average OOPs for the matched households is similar to the average OOPs of the unmatched households for inpatient care, medicines and outpatient care.

For peer review only - http://bmjopen.bmj.com/site/about/guidelines.xhtml

Table 3: Arithmetic mean OOPs by health category and questionnaire version

	Questionnaire Version-1				Questionnaire Version-2					Questionnaire Version-3				
	11 disaggregated health items				44 disaggregated health items					56 disaggregated health items				
Spending category	Number of health items aggregated	N	household average OOPs (Ghc)	SD	Number of health items aggregated	N	household average OOPs (Ghc)	SD	Estimated ratio of the means (Version-2/version-1) (95%CI)	Number of health items aggregated	N	household average OOPs (Ghc)	SD	Estimated ratio of the means (Version-3/version-1) (95%CI)
outpatient	2	81	64	135	12	181	43	130	0.70 (0.20 – 1.21)	12	105	44	78	0.75 (0.27 – 1.22)
inpatient	2	171	319	527	14	177	398	809	1.25 (0.75 – 1.74)	14	193	287	716	0.92 (0.51 – 1.34)
medicines	2	487	41	140	9	560	29	78	0.71 (0.44 – 0.98)	16	609	29	76	0.66 (0.44 – 0.88)
Preventive care	2	137	59	95	5	92	34	53	0.60 (0.33 – 0.87)	5	46	31	44	0.57 (0.27 – 0.88)
Other medical services	1	8	203	201	2	5	113	217	0.56 (-)	2	2	12	4	0.06 (-)
Health products	2	36	71	133	2	25	160	250	2.38 (-)	7	18	165	232	2.32 (-)

Note: the currency used is the Ghana cedi (GHC). US GHc4.2 was equivalent to US1$ at the time of collecting data.

Quantifying the level of agreement and variability between household and provider OOPs (matched analysis)

Overall, the household OOPs tend to be higher than the corresponding provider OOPs. We assessed the agreement between the matched household and provider OOPs first for each questionnaire version separately by estimating the overall level of agreement and variability for each type of health expenditure category. The overall bias, measured as the geometric mean ratio of household to provider OOPs saw a increasing trend with increasing numbers of health items for OOPs in only inpatient care and medicines (Table 4). There was however no evidence of an effect for outpatient or preventive care. This evidence suggests that disaggregating health expenditure items into finer specific items tended to decrease the level of agreement between matched household OOPs and corresponding provider records for inpatient care and medicines as observed in the increasing trend in the mean bias. Table 2 and Table 3 in supplementary material 1 shows details of the levels of matching for different types of providers and services received. The combine matching rate was 59%.

For peer review only - http://bmjopen.bmj.com/site/about/guidelines.xhtml

Table 4: Agreement between households and provider in OOPs by number of health expenditure items and spending category

Number of health items within each spending category	Number of households	Total number of health questions	Mean ratio	95% limits of agreement of mean ratio	Estimated difference in mean ratio between questionnaire versions & CI & p-value	Estimated diffference in SD of mean ratio between questionnaire versions & CI & p-value
	Outpatient care				p = 0.49	p = 0.50
2 health items	44	11	1.02	0.05 – 21.2	-	-
12 health items	126	44	1.21	0.05 – 26.7	1.29 (0.74 – 2.23)	1.02 (0.73 – 1.42)
12 health items	47	56	1.56	0.12 – 19.6	1.47 (0.77 – 2.80)	0.84 (0.57 – 1.25)
	Inpatient care				p = 0.003	p = 0.01
2 health items	91	11	3.88	0.17 – 86.2	-	-
14 health items	99	44	6.61	0.16 – 270.7	1.63 (0.99 – 2.69)	1.35 (1.03 – 1.76)
14 health items	100	56	9.19	0.51 – 161.2	2.34 (1.44 – 3.83)	0.93 (0.71 - 1.21)
	Medicines				p = 0.023	p = 0.33
2 health items	302	11	1.26	0.10 – 16.0	-	-
9 health items	381	44	1.35	0.09 – 19.5	1.14 (0.91 – 1.42)	1.04 (0.91 – 1.19)
16 health items	354	56	1.62	0.18 – 15.0	1.36 (1.01 – 1.70)	0.95 (0.83 – 1.08)
	Preventive care				p = 0.290	p = 0.51
2 health items	86	11	1.21	0.09 – 15.15	-	-
5 health items	67	44	0.89	0.04 – 18.9	0.74 (0.46 – 1.14)	1.19 (0.88 – 1.62)
5 health items	22	56	1.33	0.08 – 22.4	1.07 (0.55 – 2.05)	1.04 (0.68 – 1.60)

Note: The unit of the estimated difference is the mean ratio expressed as a ratio between Household and provider for each questionnaire version

We investigated why the unmatched analysis pointed to lower mean OOPs with increasing numbers of items, but the matched analysis to higher mean OOPs. There were variations in the proportions of households with matched records across type of service received by household members and type of health providers. OOPs incurred from diagnostics, the pharmacy and community health and family planning service facilities (CHPS zones) patients tended to match better than in the hospitals as observed in Table 2 of supplementary material 1. The demographic characteristics of households were similar for all households with OOPs and the matched households only (Supplementary material 3 Table 1). The mean OOPs within spending categories tended to be lower for the matched households (Supplementary material 3 Table 2). The moderate matching rates, and a

For peer review only - http://bmjopen.bmj.com/site/about/guidelines.xhtml

tendency for higher household-reported OOPs to be omitted, suggests that the matching process may have influenced the matched analysis results.

Discussion

We present evidence of the influence of the specificity of health categories on the level of agreement and variability of reported household positive OOPs by spending category. This is done by comparing household and provider data. In this study, we found that the unmatched analysis (where household data is not compared with provider data), comparing mean reported OOPs for all households, suggested a tendency for a higher number of items to result in lower positive OOPs for medicines and preventive services. This suggests that being more detailed as a consequence of increasing the number of health items within each category leads to lower mean positive OOPs in these spending categories. In assessing the agreement between matched household-reported and provider OOPs, our findings suggests that, the level of agreement between provider and household OOPs decreased with increasing specificity for medicines and inpatient care. These trends maybe attributed to the inability of households to either recall the specific name of the medicine/service received from the health provider or they did not know the name of the medicine/service at all when such information is required at a very detailed level. Another potential factor could be a possible introduction of bias by the matching process in our study, with the possibility that smaller amounts matched better with provider data than larger amounts. These factors may require further investigations beyond the scope of this current study.

A number of studies have investigated and documented the potential effect and consequence of varying number of health items on the estimation of OOPs using nationally representative survey data [8,12,13,23]. All these studies have mostly focused their investigations on the effect of number of health items on total household health expenditure without examining the effect by spending categories or distinguishing between comprehensiveness of the health expenditure list (number of health categories covered) from specificity of each category (number of questions per health category) therefore making such studies deficient in identifying the most reliable and accurate survey tools.

The primary purpose of this study is to investigate the effect of the specificity of the health expenditure question on both the total amount spent on health out-of-pocket and amounts spent by type of service or medical products. While the total amount is a critically needed to assess to what extent countries rely on households' direct contributions to fund the health system within the national health account framework on the one hand and their impact on household's welfare in the context of financial protection monitoring, information on levels by type of spending categories are critical to better inform policies. It is important to know if households are mostly contributing to fund medicines versus inpatient care to give just one example.

For peer review only - http://bmjopen.bmj.com/site/about/guidelines.xhtml

Hence, knowing that the total level of OOPs is over or under-estimated is insufficient and understanding which type of expenditure is driving such over or under-estimation is critical.

This paper is the first one to have attempted to shed light on this particular aspect. Others have focused on either one specific category (e.g inpatient care) or completely ignored this issue [13] in most cases relying on cross-sectional comparisons across multiple countries that also differ in terms of the choice of the recall period and total number of non-medical questions (4,11–13).

By using an experimental design we were able to control for other potential confounders (recall period, number of non-medical expenditure question, type of survey). We also aimed at validating households' responses. The challenges faced during the implementation impacted in our initial plan in two ways: first we are not able to validate the total amount spent on health out-of-pocket on all types of good and services. Second, we had to restrict the validation part to those households that reported a health expenditure (the spenders). Despite these limitations, this paper remains the first one to attempt validation on multiple type of spending and we are able to show that the accuracy of different type of services/products is differently affected

Conclusion

This study describes a novel validation study in a rural setting that operates a demographic surveillance system. The evidence from this study suggests caution in interpreting OOPs from different survey instruments relying on different levels of detail per spending category even in the absence of differences in recall period and other survey questionnaire design features. We found that systematically decomposing health expenditure items into more specific and finer sub-classes leads to lower average OOPs for outpatient care, medicines and preventive care when comparing across different version without comparing to provider records. Validation studies need to consider the possibility of bias introduced by the matching approaches.

For peer review only - http://bmjopen.bmj.com/site/about/guidelines.xhtml

List of abbreviations

DHS Demographic and Health Survey

NHRC Navrongo Health Research Centre

NHDSS Navrongo Health and Demographic Surveillance System

GLSS Ghana Living Standards Survey

COICOP Classification of Individual Consumption according to purpose.

iHOPE INDEPTH-Network Household Out-of-pocket Expenditure

HHS Household Health Survey

SHA System of Health Accounts

NHA National Health Accounts

NHIS National Health Insurance Scheme

OOPs Out-of-Pocket Health Spending

LMIC Low and Middle-income Countries

LSMS Living Standards Measurement Survey

WHO World Health Organization

WHS World Health Survey

Consent for publication

Written informed consent was obtained from all patients for publication of this manuscript.

Availability of data and material

Extra data can be accessed via the Dryad data repository at http://datadryad.org/ with the doi:10.5061/dryad.nk98sf7sw

Competing interests

The authors declare that they have no competing interests.

Funding

This project was jointly funded by the INDEPTH-Network in Accra and the Swiss tropical and public health institute of the University Of Basel, Switzerland through a grand from Bill and Melinda gates foundation, grant number OPP1113162. GF, TE, AR were partially supported by WHO.

Author contributions

The research protocol was developed by TE, GF, JA, FT

For peer review only

The research protocol was written by GF, TE, JA, FT, YDB

The questionnaires were developed by GF, IAA

The analytical plan was developed by AR, IAA, LML, GF

Data collection: IAA, MD, NM, AO

Data cleaning: IAA, NM, MD, JS

Data analysis: IAA, AR

First draft was written by: IAA, AR

All authors contributed to writing of the manuscript

Acknowledgements

The authors wish to thank all the study participants, health facilities and the entire iHOPE Ghana team for participating in this study. We are very grateful to INDEPTH-Network and Swiss Topical and Public Health Institute for their technical support during the entire phase of this study. We also appreciate the financial support from the Bill and Melinda Gates foundation for the study.

For peer review only - http://bmjopen.bmj.com/site/about/guidelines.xhtml

References

1. SHA. A System of Health Accounts [Internet]. OECD Publishing; 2011 [cited 2017 Jul 12]. Available from: http://www.oecd-ilibrary.org/social-issues-migration-health/a-system-of-health-accounts_9789264116016-en

2. O'Donnell O, van Doorslaer E, Wagstaff A, Lindelow M. Analyzing Health Equity Using Household Survey Data: A Guide to Techniques and their Implementation [Internet]. The World Bank; 2007 [cited 2019 Feb 26]. Available from: http://elibrary.worldbank.org/doi/book/10.1596/978-0-8213-6933-3

3. O'Donnell O. Financial protection against medical expense. Tinbergen Inst Discuss Pap. 2019 Jan;47.

4. Xu K, Evans DB, Kawabata K, Zeramdini R, Klavus J, Murray CJ. Household catastrophic health expenditure: a multicountry analysis. The Lancet. 2003 Jul 12;362(9378):111–7.

5. Wagstaff A, Flores G, Smitz M-F, Hsu J, Chepynoga K, Eozenou P. Progress on impoverishing health spending in 122 countries: a retrospective observational study. Lancet Glob Health. 2018 Feb;6(2):e180–92.

6. Thomson S, Cylus J, Evetovits T, Srakar A. Can people afford to pay for health care? new evidence on financial protection in Europe: [regional report [Internet]. Copenhagen: World Health Organization, Regional Office for Europe; 2019 [cited 2019 Oct 14]. Available from: https://apps.who.int/iris/bitstream/handle/10665/311654/9789289054058-eng.pdf?sequence=1&isAllowed=y

7. Rannan-Eliya RP, Lorenzoni L. Guidelines for Improving the Comparability and Availability of Private Health Expenditures Under the System of Health Accounts Framework [Internet]. 2010 May [cited 2019 Feb 24]. Report No.: 52. Available from: https://www.oecd-ilibrary.org/social-issues-migration-health/guidelines-for-improving-the-comparability-and-availability-of-private-health-expenditures-under-the-system-of-health-accounts-framework_5kmbrcg0clvc-en

8. Lu C, Chin B, Li G, Murray CJ. Limitations of methods for measuring out-of-pocket and catastrophic private health expenditures. Bull World Health Organ. 2009 Mar;87(3):238–44.

9. Neter J, Waksberg J. A Study of Response Errors in Expenditures Data from Household Interviews. J Am Stat Assoc. 1964 Mar 1;59(305):18–55.

10. Neter J. Measurement Errors in Reports of Consumer Expenditures. J Mark Res. 1970;7(1):11–25.

11. Heijink R, Xu K, Saksena P, Evans D. Validity and Comparability of Out-of-pocket Health Expenditure from Household Surveys: A review of the literature and current survey instruments [Internet]. World Health Organization; 2011 [cited 2019 Feb 24]. Available from: https://www.who.int/health_financing/documents/dp_e_11_01-oop_errors.pdf?ua=1

12. Xu K, Ravndal F, Evans DB, Carrin G. Assessing the reliability of household expenditure data: Results of the World Health Survey. Health Policy. 2009 Aug;91(3):297–305.

13. Lavado RF, Brooks BP, Hanlon M. Estimating health expenditure shares from household surveys. Bull World Health Organ. 2013 Jul 1;91(7):519-524C.

14. GLSS6 Report. Ghana Living Standards Survey Round 6 (GLSS6) Main Report [Internet]. Ghana Statistical Service; 2014 Aug [cited 2019 Oct 11] p. 244. Report No.: 6. Available from: http://www.statsghana.gov.gh/gssmain/fileUpload/Living%20conditions/GLSS6_Main%20Report.pdf

For peer review only - http://bmjopen.bmj.com/site/about/guidelines.xhtml

15. United Nations. Classification of Individual Consumption According to Purpose (COICOP) 2018. U N. 2018;265.

16. Oduro AR, Wak G, Azongo D, Debpuur C, Wontuo P, Kondayire F, et al. Profile of the Navrongo Health and Demographic Surveillance System. Int J Epidemiol. 2012 Aug 1;41(4):968–76.

17. Leive A. Coping with out-of-pocket health payments: empirical evidence from 15 African countries. Bull World Health Organ. 2008 Nov 1;86(11):849–56.

18. Addae-korankye alex. Challenges of Financing Health Care in Ghana: The Case of National Health Insurance Scheme (NHIS). In 2013.

19. Wang H, Otoo N, Dsane-Selby L. Ghana National Health Insurance Scheme. 2017;71.

20. Bland JM. Sample size for a study of agreement between two methods of measurement [Internet]. 2004 [cited 2019 Feb 28]. Available from: https://www-users.york.ac.uk/~mb55/meas/sizemeth.htm

21. Giavarina D. Understanding Bland Altman analysis. Biochem Medica. 2015 Jun 5;25(2):141–51.

22. Bland JM, Altman DG. Measuring agreement in method comparison studies. 1999;(Statistical Methods in Medical Research):26.

23. Grosh EM, Glewwe P. Designing Household Survey Questionnaires for Developing Countries: Lessons from Ten Years of LSMS Experience. :78.

Figure Legend

Figure 1: flow chart of household enrolment and matching to provider records

For peer review only

Figure 1: flow chart of household enrolment and matching to provider records

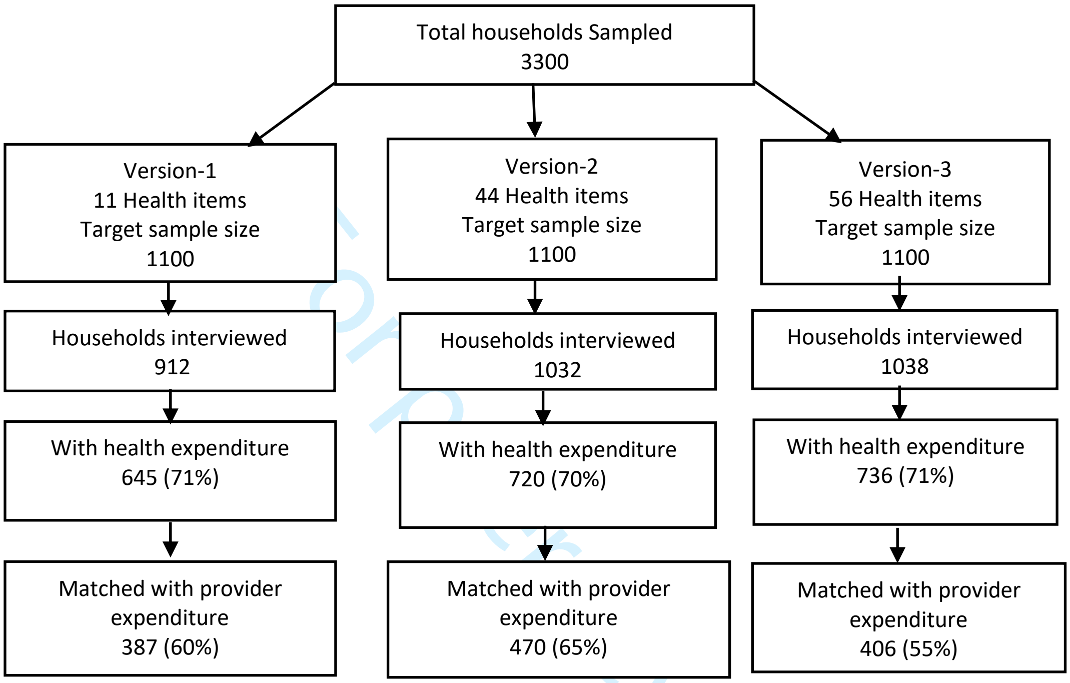

For peer review only - http://bmjopen.bmj.com/site/about/guidelines.xhtml

Supplementary material 1

Data collection instrument design and matching procedure

Ghana Living Standards Survey 6 (GLSS6)

The Ghana Living Standards survey (GLSS) is a multi-purpose household survey instituted by the World Bank to collects information on many different dimensions of living conditions, including education, health, employment and household expenditure on food and non-food items. The GLSS 6 is the sixth edition of the survey conducted in 2012/2013 (GLSS6 Report, 2014). The survey instrument has one large module on household consumption reported by a single respondent and structured around food versus non-food expenditures (health expenditure included) using similar recall periods for all food and non-food divisions regardless of their relevance in gathering information on health. The consumption module in the survey is comprehensive in covering household consumption items. The structure of the consumption module follows the Classification of individual Consumption According to Purpose (COICOP 1999) classifications. COICOP-1999 is the international reference classification of household expenditure which aims at providing a framework of homogeneous categories of goods and services considered a function or purpose of household consumption expenditure. Following this classification, the GLSS6 has 251 items in the consumption module, of these; about 7% (17 items) are questions asking about health expenditure. The GLSS6 adopted the second level of disaggregation of COICOP-1999 for collecting information on Health expenditure in the consumption module but COICOP-1999 was of little guidance when there is an interest in refining the information on health expenditure beyond the second level of disaggregation. Previous analysis (Lavado, Brooks & Hanlon, 2013) has shown that as the total number of health and non-health consumption expenditure question increases, the average health expenditure budget share tends to either decrease or increase and as such given the structure of the GLSS, it is difficult to assess the accuracy of the information collected. The GLSS has been the primary source of data for estimating OOPs in Ghana.

Specificity of the iHOPE household survey instrument

The starting point to develop questions on OOPs was the GLSS6 but with the objective to align it with division 6 of COICOP-2018 which was refined to better cover all the different type of health products and services available to the population. Version 1 of the OOPs questionnaire captured six major categories namely medicines, health products, preventive care services, other outpatient care services, inpatient care services and other health services to obtain a total of 11 health items. The second questionnaire further expanded all main six categories (Table 1) to a total of 44 health items. In the third questionnaire version only questions on OOPs for medicines and health products were expanded to arrive at a total of 56 health items for version three. Main difference between questions in OOPs included in the GLSS6 and those developed for this study are listed in Table 1.

For peer review only

The new developed questions on out-of-pocket health care expenditure were then included at the end of GLSS6 consumption module. Questions related to the provider were asked to each person reporting an OOP in order to be able to compare provider and household information. The final instruments included in addition to the consumption expenditure module a household roster, a module on housing characteristics and household assets. Figure 1 shows the generic structure of the developed instrument.

Figure 1: Structure of survey instruments

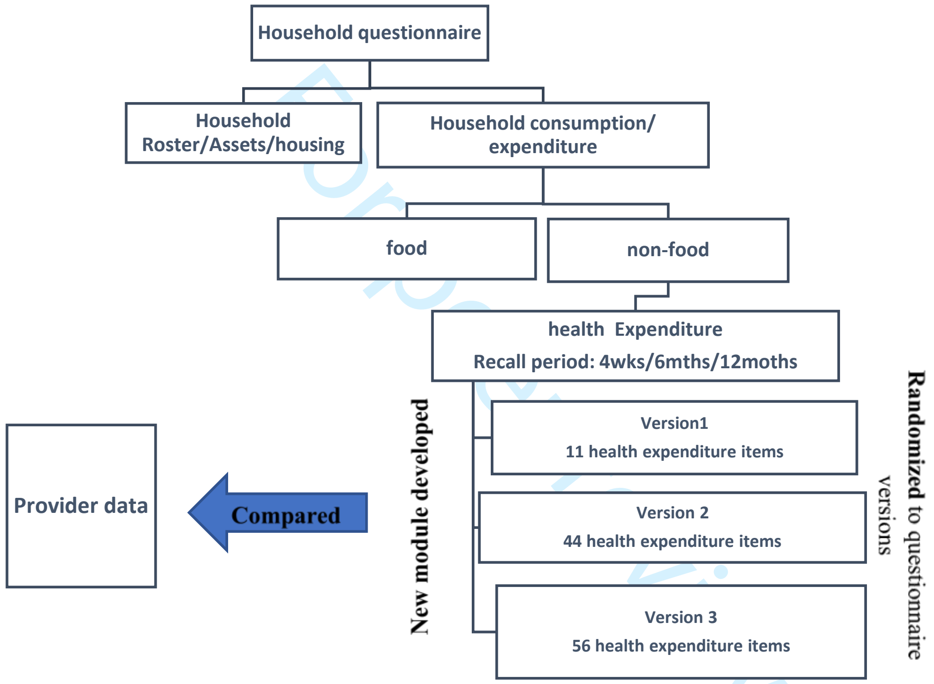

For peer review only

Table 1: Specificity of the health expenditure list

Table 1GLSS 6	Version 1	Version 2	Version 3
Based on COICOP-1999, cover all major classes of health products and services.	covers all major classes of health products and services are redefined in COICOP-2018		
Total number of questions on OOPs for health is 24 split as follows:	Total number of questions on OOPs for health is 11 split as follows:	Total number of questions on OOPs for health is 44. Expand version 1 as follows:	Total number of questions on OOPs for health is 56. Expand version 2 as follows:
Pharmaceutic products and other medical products – 6 questions	Medicines – 2 questions to distinguish herbal from non-herbal medicine	Expands questions on non-herbal medicines (7 questions)	Questions on non-herbal medicines are split into prescribed and non-prescribed medicines
MEDICAL PRODUCTS, APPLIANCES AND EQUIPMENT – 2 questions to differentiate appliance and equipment from assistive products	Health products – 2 questions to distinguish assistive products from an overall category on medical diagnostic products, prevention and protective devices.	Differentiates between purchase of assistive products and repair/rental/maintenance of assistive products	Within purchase and repair of assistive products a distinction is made between those for vision and hearing versus mobility and daily living. The overall category on medical diagnostic products, prevention and protective devices is split into 3.
Outpatient medical services – 14 questions to differential type of health care provider and public/private	Outpatient medical services – 4 questions to differentiate within preventive services immunization/vaccination services from other preventive services (e.g. check-ups); dental outpatient services from other outpatient services.	Immunization vaccination services differentiate between those related to maternal and child care from other vaccination services.<br><br>Other preventive services are split into 3 categories<br>Dental care services and other outpatient services are split by type of facility (hospital versus outpatient setting) and OOPs for consultation are differentiated from OOPs for laboratory tests, imagining services when possible. In addition for other outpatient services the type of health care provider is also distinguished (e.g. specialist, general doctor)	
Hospital services – 2 questions to distinguish public/private	Inpatient services – 2 questions to differentiate long term inpatient care from other overnight stays	Details on inpatient long term care and other overnight stays are gathered by the means of	
	Other health services - 1 question to capture emergency transportation and emergency rescue services	Emergency transportation is split into 2 categories 7 questions covering fees, products, tests and emergency transportation related to the inpatient treatment	

Provider data collections

Within the Ghana health care system, public health providers who are managed by the government keep patient records as part of routine activities while most private providers either kept minimal transactional records or no records at all. To generate accurate data for comparison, we developed a template (Supplementary material 2) and trained private provider owners on how to use it to collect patient information. The main fields in the template included name, address, phone number, referral status, reason for consultation and cost of treatment/service. For public providers where patient data was already

recorded, trained field workers completed the template by review and extracting relevant information from the provider records. We therefore purposely selected major health care providers within the demographic surveillance site to obtain the provider data. The criterion for selecting the providers was based on the availability of transactional data or a care-taker who could record details of the transactions from clients in the case of private providers. The public health providers selected include; one hospital, one clinic and seven public health centres. For the private health care providers, ten high volume pharmacy and license chemical shops met our selection criteria and were selected.

Matching

Matched samples in this study refer to households that were linked to their provider records. For any households that reported positive expenditure on any of the health expenditure items, corresponding health provider data was obtained from the provider records using details about the provider obtained from the respondents. The linked household-provider data formed a matched sample used in our analysis. The matching of household and provider data was done at the individual household member level and by spending category. Due to challenges of completeness and accuracy of provider details provided by respondents and possible errors in recording patient details at the provider, some households could not be matched to provider data and were therefore excluded from the analysis. The challenges that influenced the completeness and accuracy of provider data are discussed in another paper "Challenges in linking household health expenditure records to provider records" (Agorinya et al, in prep)

Table 2: OOPs that successfully matched with health provider records by type of health provider

Type of provider	Total number of clients attending provider	proportion of clients with linked records to household
Hospital	453	46.8
Community Health Centre	195	55.4
CHPS	196	90.3
Clinic	58	27.6
Chemical Shop	194	71.1
Diagnostic laboratory	29	82.8
Hawker/Vendor/ Mobile Van	25	0.0
General local shop	81	33.3
Other	16	12.5
Pharmacy	155	73.6
Total	1402	59

Table 3: OOPs that successfully matched with health provider records by type of care received

Spending category	Total number of cases	Number of cases matched	Proportion of cases matched
Inpatient care	339	159	47.2
Out-patient	551	351	63.4
Medicines	468	286	61.6
Preventive care	32	22	68.8
Medical products	7	1	14.3
Total	1397	820	59

For peer review only

Supplementary material 2

COICOP 2018 based Levels of disaggregation of Health items

Type of information	*Version-1*<br>**Level of *D*isaggregation – 11 Health items**	Recall periods:<br>D- days/ M-Months				
		**15d**	**30d**	**3M**	**6M**	**12M**
	**COICOP code 06.2.3 Inpatient care services**					
**To be asked in bold**<br>Examples are given in brackets for more see explanatory notes.<br>**If helpful can add at the end of the explanations: for patients with disabilities, the elderly (or those who requires permanent surveillance or constant help due to limited functional capacity)**	**I.LONG. Medical treatment and / or care that required overnight stay in a nursing home; (medical convalescent homes; palliative care establishments) or any other long term care medical facility**				X	X
	**I.CURR. Medical and dental treatment that required an overnight stay in any type of facility** (e.g. hospitals, clinics) **excluding long term care medical facility**				X	X
**Inclusion/Exclusion criteria to be specified when asking about the amount**<br>Applies to I.LONG & I.CURR	Includes payments for all medical services, diagnostic and laboratory tests, medicines and medical products needed during the overnight stay. Also include emergency transportation services and emergency rescue.<br>Excludes: non-emergency transportation and non-medical costs for patient's relative.					
	**COICOP code 06.2.1 Preventive care services**					
	**P.IMMV. Immunization/vaccination services including for maternal and child care**			X	X	
**Inclusion/Exclusion criteria to be specified when asking about the amount**	Includes ; travel and tourism vaccination as well as any other immunization/vaccination service.					
	**P.OTHR. Other preventive services such as prenatal/postnatal care, child growth and development visits, family planning, screening, tests, consultations to detect communicable or non-communicable diseases before symptoms appear** (e.g. diabetes, heart problems)			X	X	
**Inclusion/Exclusion criteria to be specified when asking about the amount**<br>Applies to both P.IMMV & P.OTHR	Includes diagnostic and laboratory tests needed to provide preventive services but exclude payments for the vaccine itself when separately invoiced from the service.					
	**COICOP code 06.4.2 Emergency transportation and emergency rescue**					
**Alternative wording: transportation for medical emergency reasons (e.g. by ambulance)**	**O.EMER. Patient emergency transportation services and emergency rescue services (excluding those associated with an overnight stay)**	X	X			
**Inclusion/Exclusion criteria to be specified when asking about the amount**	Excludes non-emergency transportation services					
	**COICOP code 06.2.2 & 06.2.3 Outpatient dental & other outpatient services**					
	**O.DENT. Dental consultations and services that did not require an overnight stay;**	X	X			
**Inclusion/Exclusion criteria to be specified when asking about the amount**	Includes dental diagnostics services and laboratory tests needed to provide outpatient dental services (e.g. X-rays, blood tests)<br>For any dental illness, disease, injury or health problem; from any type of provider; inside or outside a hospital setting					
	**O.CRRL. other medical consultations and services than dental and preventive that did not require an overnight stay**	X	X			
**Inclusion/Exclusion criteria to be specified when asking about the amount**	Includes any diagnostic and laboratory test needed to provide outpatient medical services (e.g. X-rays, blood/urine tests), but excludes emergency transportation services and emergency rescue					
	**COICOP code 06.1.1**					
	**M.HERH. Herbal medicines (tablets or syrups) and homeopathic products for consumption outside a health facility or institution.**	X	X			
	**M.MVCP. Medicines** (branded, generic)**, vaccines, oral contraceptives, vitamins and minerals and other pharmaceutical preparations for consumption outside a health facility or institution.**	X	X			
	**COICOP code 06.1. 2 medical diagnostic products, prevention and protective devices**					
**Inclusion/Exclusion criteria to be specified when asking about the amount**	**D.** (Pregnancy tests, incontinence products and absorbent including diapers for the aging population, inhalers, mechanical contraceptives; insecticide treated mosquito nets, blood pressure devices) **and other medical health products for personal use outside a health facility or institution.**	X	X			
Only applies to some diagnostic products	Includes repair, rental and maintenance					
	**COICOP code 06.1.3 Assistive products for vision, hearing, mobility and daily living.**					

	**A. Purchase, repair, rental/maintenance of** (glasses for vision; hearing aids; crutches & wheelchairs; therapeutic footwear; walkers; pressure relief mattresses) **and all other assistive health products** .				X	X
**Type of information**	***Version-2***<br>**Level of *D*isaggregation – 44 Health items**	**Recall periods: D- days/ M-Months**				
		**15d**	**30d**	**3M**	**6M**	**12M**
	**COICOP code 06.2.3 Inpatient care services**					
**To be asked in bold**<br>Examples are given in brackets for more see explanatory notes<br>**If helpful can add at the end of the explanations: for patients with disabilities, the elderly (or those who requires permanent surveillance or constant help due to limited functional capacity)**	**I.LONG. Medical treatment and / or care that required overnight stay in a nursing home; (medical convalescent homes; palliative care establishments) or any other long term care medical facility**<br>**If yes**					
	**I.LONG.SP.1 medical services during the overnight long term care**				X	X
	**I.LONG.SP.2 medicines during the overnight long term care**				X	X
	**I.LONG.SP.3 medical products during the overnight long term care**				X	X
	**I.LONG.DT. diagnostic and laboratory tests**				X	X
	**I.LONG.NM. Non-medical cost for the patient (cooking, cleaning, accommodation) during the overnight long term care**				X	X
	**I.LONG.ER.1 Emergency transportation and rescue services by ambulance or other vehicles specially adjusted for medical purposes**				X	X
	**I.LONG.ER.2 Emergency transportation services and rescue by ordinary vehicles or airplanes (not specially adjusted for a medical purpose)**				X	X
	**I.CURR Medical and dental treatment that required an overnight stay in any type of facility** (e.g. hospitals, clinics) **excluding long term care medical facility**<br>**If yes**					
	**I.CURR.SP.1. medical, dental services during overnight stay**				X	X
	**I.CURR.SP.2. medicines for medical or dental treatment during overnight stay**				X	X
	**I.CURR.SP.3. medical products for medical or dental treatment during overnight stay**				X	X
	**I.CURR.DT. diagnostic and laboratory tests for medical or dental treatment during overnight stay (e.g. x-rays, scans, blood tests)**				X	X
	**I.CURR.NM. Non-medical costs for the patient (cooking, cleaning, accommodation)**				X	X
	**I.CURR.ER.1. emergency transportation and rescue services by ambulance or other vehicles specially adjusted for medical purposes**				X	X
	**I.CURR.ER.2. Emergency transportation services and rescue by ordinary vehicles or airplanes (not specially adjusted for a medical purpose)**				X	X
**Inclusion/Exclusion criteria to be specified when asking about the amount**<br>Applies to both I.LONG.SP.1 & I.CURR.SP.1	*If possible* exclude diagnostic and laboratory tests during the overnight stay I.LONG.DT/ I.CURR.DT. and emergency transportation services and emergency rescue (I.LONG.ER / I.CURR.ER)					
Applies to all I.LONG. & I.CURR	Excludes: non-emergency transportation and non-medical costs for patient's relative.					
	**COICOP code 06.2.1 Preventive care services**					
	**P.IMMV.MC. Immunization/vaccination services for maternal and child care**			X	X	
	**P.IMMV.OV. Travel and tourism vaccination, any other compulsory or voluntary immunization/vaccination service.**			X	X	
**Inclusion/Exclusion criteria to be specified when asking about the amount**<br>Applies to P.IMMV.OV	Includes; travel and tourism vaccination as well as any other immunization/vaccination service.					
Applies to both P.IMMV.MC and P.IMMV.OV	Excludes payments for the vaccine itself when separately invoiced from the service.					
	**P.OTHR. Other preventive services than immunization/vaccination**					
	**P.OTHR.GH.1 Family planning, counselling, prenatal/postnatal care services for both the mother and new born (during the six weeks or 42 days)**			X	X	
	**P.OTHR.GH.2 Child growth and development consultation visits and any other consultations to monitor "good" health of children and adults**			X	X	
	**P.OTHR.DI. screening, tests, consultations to detect communicable or non-communicable diseases before symptoms appear** (e.g. diabetes, heart problems)			X	X	
**Inclusion/Exclusion criteria to be specified when asking about the amount**<br>Applies to all P.IMMV & P.OTHR	Includes diagnostic and laboratory tests needed to provide preventive services					
	**COICOP code 06.4.2 Emergency transportation and emergency rescue**					
**Alternative wording: transportation for medical emergency reasons (e.g. by ambulance)**	**O.EMER. Patient emergency transportation services and emergency rescue services (excluding those associated with an overnight stay)**					

	**O.EMER.AV. by ambulance or other vehicles specially adjusted for medical purpose**	X	X			
	**O.EMER.NA. by ordinary vehicles or airplanes (not specially adjusted for a medical purpose)**	X	X			
**Inclusion/Exclusion criteria to be specified when asking about the amount**	Excludes non-emergency transportation services					
	**COICOP code 06.2.2 & 06.2.3 Outpatient dental & other outpatient services**					
	**O.DENT Outpatient dental care**					
	**In a hospital setting**					
	**O.DENT.CS.1 Dental consultations and services that did not require an overnight stay in a hospital setting**	X	X			
	**O.DENT.DT.1. Diagnostic and laboratory tests needed to provide dental consultations and services in a hospital setting**	X	X			
	**Outpatient settings (e.g. private practice, office, medical center, clinics, polyclinics)**					
	**O.DENT.CS.2. Dental consultations and services that did not require an overnight stay Outpatient settings (e.g. private practice, office, medical center, clinics, polyclinics)**	X	X			
	**O.DENT.DT.2. Diagnostic and laboratory tests needed to provide dental consultations and services Outpatient settings (e.g. private practice, office, medical center, clinics, polyclinics)**	X	X			
**Inclusion/Exclusion criteria to be specified when asking about the amount** Applies to O.DENT.CS..1 & O.DENT.CS.2	Excludes dental diagnostics services and laboratory tests needed to provide outpatient dental services (e.g. X-rays, blood tests)					
	**O.CRRL. Other medical consultations and services than dental and preventive that did not require an overnight stay**					
	**In a hospital setting**					
	**O.CRRL.CS.1.1 consultations and services of specialists (paediatricians, surgeons, cardiologists, ophthalmologist, mental health)**	X	X			
	**O.CRRL.CS.1.2 consultation and services of general doctors**	X	X			
	**O.CRRL.CS.1.3 consultation and services of nurses, midwifes and other health care practitioner**	X	X			
	**O.CRRL.DT.1 diagnostic and laboratory tests needed to provide other medical services that did not require an overnight stay in a hospital setting**	X	X			
	**Outpatient settings (e.g. private practice, office, medical center, clinics, polyclinics)**					
	**O.CRRL.CS.2.1 consultations and services of specialists (paediatricians, surgeons, cardiologists, ophthalmologist, mental health)**	X	X			
	**O.CRRL.CS.2.2 consultation and services of general doctors**	X	X			
	**O.CRRL.CS.2.3 consultation and services of nurses, midwifes and other health care practitioner**	X	X			
	**O.CRRL.DT.2 diagnostic and laboratory tests needed to provide other medical services that did not require an overnight stay outside a hospital setting**	X	X			
**Inclusion/Exclusion criteria to be specified when asking about the amount** Applies to all O.CRRL.CS.1 & O.CRRL.CS.2	excludes diagnostic and laboratory test needed to provide outpatient medical services (e.g. X-rays, blood/urine tests)					
	**COICOP code 06.1.1**					
	**M.HERH. Herbal medicines (tablets or syrups) and homeopathic products for consumption outside a health facility or institution.**	X	X			
	**M.MVCP. Medicines** (branded, generic)**, vaccines, oral contraceptives, vitamins and minerals for consumption outside a health facility or institution.**					
	**M.MVCP.IA. antibiotics**	X	X			
	**M.MVCP.IO.Other medicines (branded, generic, homeopathic) to treat (presumed or diagnosed) bacterial infections (e.g. malaria, diarrhoeas, dysentery, increased frequency of stools with or without blood and mucus in stools; worms infestations)**	X	X			
	**M.MVCP.CD. medicines to treat (presumed or diagnosed) non-communicable diseases or chronic diseases** (e.g. diabetes, hypertension)	X	X			
	**M.MVCP.FP. oral contraceptives and contraceptives in the form of injections**	X	X			
	**M.MVCP.SY medicines to treat fevers, pain and other symptoms (e.g. nausea; vomiting, constipation; inflammation)**	X	X			
	**M.MVCP.VM. vitamins, mineral**	X	X			
	**M.MVCP.OM. other medicines and pharmaceutical preparations not elsewhere specified**	X	X			
	**COICOP code 06.1. 2 medical diagnostic products, prevention and protective devices**					

For peer review only

	**D.** (Pregnancy tests, incontinence products and absorbent including diapers for the aging population, inhalers, mechanical contraceptives; insecticide treated mosquito nets, blood pressure devices) **and other medical health products for personal use outside a health facility or institution**.	X	X			
Inclusion/Exclusion criteria to be specified when asking about the amount<br>Only applies to some diagnostic products	Includes repair, rental and maintenance					
	**COICOP code 06.1.3 Assistive products for vision, hearing, mobility and daily living.**					
	**A.PURC. Purchase of** (glasses for vision; hearing aids; crutches & wheelchairs; therapeutic footwear; walkers; pressure relief mattresses) **and all other assistive health products** .				X	X
	**A.RRMN. Repair, rental/maintenance of** (glasses for vision; hearing aids; crutches & wheelchairs; therapeutic footwear; walkers; pressure relief mattresses) **and all other assistive health products** .				X	X

For peer review only

Type of information	*Version-3*<br>Level of Disaggregation – 56 Health items	Recall periods:<br>D- days/ M-Months				
		15d	30d	3M	6M	12M
	<u>COICOP code 06.2.3 Inpatient care services</u>					
**<u>To be asked in bold</u>**<br>Examples are given in brackets for more see explanatory notes<br>**If helpful can add at the end of the explanations: for patients with disabilities, the elderly (or those who requires permanent surveillance or constant help due to limited functional capacity)**	**I.LONG. Medical treatment and / or care that required overnight stay in a nursing home; (medical convalescent homes; palliative care establishments) or any other long term care medical facility**<br>**If yes**					
	**I.LONG.SP.1 medical services during the overnight long term care**				X	X
	**I.LONG.SP.2 medicines during the overnight long term care**				X	X
	**I.LONG.SP.3 medical products during the overnight long term care**				X	X
	**I.LONG.DT. diagnostic and laboratory tests**				X	X
	**I.LONG.NM. Non-medical cost for the patient (cooking, cleaning, accommodation) during the overnight long term care**				X	X
	**I.LONG.ER.1 Emergency transportation and rescue services by ambulance or other vehicles specially adjusted for medical purposes**				X	X
	**I.LONG.ER.2 Emergency transportation services and rescue by ordinary vehicles or airplanes (not specially adjusted for a medical purpose)**				X	X
	**Medical and dental treatment that required an overnight stay in any type of facility** (e.g. hospitals, clinics) **excluding long term care medical facility**<br>**If yes**					
	**I.CURR.SP.1. medical, dental services during overnight stay**				X	X
	**I.CURR.SP.2 medicines for medical or dental treatment during overnight stay**				X	X
	**I.CURR.SP.3 medical products for medical or dental treatment during overnight stay**				X	X
	**I.CURR.DT. diagnostic and laboratory tests for medical or dental treatment during overnight stay (e.g. x-rays, scans, blood tests)**				X	X
	**I.CURR.NM. Non-medical costs for the patient (cooking, cleaning, accommodation)**				X	X
	**I.CURR.ER.1.emergency transportation and rescue services by ambulance or other vehicles specially adjusted for medical purposes**				X	X
	**I.CURR.ER.2. Emergency transportation services and rescue by ordinary vehicles or airplanes (not specially adjusted for a medical purpose)**				X	X
**<u>Inclusion/Exclusion criteria to be specified when asking about the amount</u>**<br>Applies to both I.LONG.SP.1 & I.CURR.SP.1	*If possible* exclude diagnostic and laboratory tests during the overnight stay I.LONG.DT/ I.CURR.DT. and emergency transportation services and emergency rescue (I.LONG.ER / I.CURR.ER)					
Applies to all I.LONG. & I.CURR	Excludes: non-emergency transportation and non-medical costs for patient's relative.					
	<u>COICOP code 06.2.1 Preventive care services</u>					
	**P.IMMV.MC Immunization/vaccination services for maternal and child care**			X	X	
	**P.IMMV.OV Travel and tourism vaccination, any other compulsory or voluntary immunization/vaccination service.**			X	X	
**<u>Inclusion/Exclusion criteria to be specified when asking about the amount</u>**<br>Applies to P.IMMV.OV	Includes; travel and tourism vaccination as well as any other immunization/vaccination service.					
Applies to both P.IMMV.MC and P.IMMV.OV	Excludes payments for the vaccine itself when separately invoiced from the service.					
	**P.OTHR. Other preventive services than immunization/vaccination**					
	**P.OTHR.GH.1 Family planning, counselling, prenatal/postnatal care services for both the mother and new born (during the six weeks or 42 days)**			X	X	
	**P.OTHR.GH.2 Child growth and development consultation visits and any other consultations to monitor "good" health**			X	X	
	**P.OTHR.DI. screening, tests, <u>consultations</u> to detect communicable or non-communicable diseases before symptoms appear** (e.g. diabetes, heart problems)			X	X	
**<u>Inclusion/Exclusion criteria to be specified when asking about the amount</u>**<br>Applies to all P.IMMV & P.OTHR	Includes diagnostic and laboratory tests needed to provide preventive services					
	<u>COICOP code 06.4.2 Emergency transportation and emergency rescue</u>					
**Alternative wording: transportation for medical emergency reasons (e.g. by ambulance)**	**O.EMER. Patient emergency transportation services and emergency rescue services (excluding those associated with an overnight stay)**					

	**O.EMER.AV by ambulance or other vehicles specially adjusted for medical purpose**	X	X			
	**O.EMER.NA by ordinary vehicles or airplanes (not specially adjusted for a medical purpose)**	X	X			
**Inclusion/Exclusion criteria to be specified when asking about the amount**	Excludes non-emergency transportation services					
	**COICOP code 06.2.2 & 06.2.3 Outpatient dental & other outpatient services**					
	**O.DENT Outpatient dental care**					
	**O.DENT.CS.1 Dental consultations and services that did not require an overnight stay in a hospital setting**	X	X			
	**O.DENT.DT.1 Diagnostic and laboratory tests needed to provide dental consultations and services in a hospital setting**	X	X			
	**O.DENT.CS.2 Dental consultations and services that did not require an overnight stay Outpatient settings (e.g. private practice, office, medical center, clinics, polyclinics)**	X	X			
	**O.DENT.DT.2 Diagnostic and laboratory tests needed to provide dental consultations and services Outpatient settings (e.g. private practice, office, medical center, clinics, polyclinics)**	X	X			
**Inclusion/Exclusion criteria to be specified when asking about the amount** Applies to O.DENT.CS.1 & O.DENT.CS.2	Excludes dental diagnostics services and laboratory tests needed to provide outpatient dental services (e.g. X-rays, blood tests)					
	**O.CRRL. Other medical consultations and services than dental and preventive that did not require an overnight stay**					
	**In a hospital setting**					
	**O.CRRL.CS.1.1 consultations and services of specialists (paediatricians, surgeons, cardiologists, ophthalmologist, mental health)**	X	X			
	**O.CRRL.CS.1.2 consultation and services of general doctors**	X	X			
	**O.CRRL.CS.1.3 consultation and services of nurses, midwifes and other health care practitioner**	X	X			
	**O.CRRL.DT.1 diagnostic and laboratory tests needed to provide other medical services that did not require an overnight stay in a hospital setting**	X	X			
	**Outpatient settings (e.g. private practice, office, medical center, clinics, polyclinics)**					
	**O.CRRL.CS.2.1 consultations and services of specialists (paediatricians, surgeons, cardiologists, ophthalmologist, mental health)**	X	X			
	**O.CRRL.CS.2.2 consultation and services of general doctors**	X	X			
	**O.CRRL.CS.2.3 consultation and services of nurses, midwifes and other health care practitioner**	X	X			
	**O.CRRL.DT.2. diagnostic and laboratory tests needed to provide other medical services that did not require an overnight stay outside a hospital setting**	X	X			
**Inclusion/Exclusion criteria to be specified when asking about the amount** Applies to all O.CRRL..CS1 & O.CRRL..CS.2	excludes diagnostic and laboratory test needed to provide outpatient medical services (e.g. X-rays, blood/urine tests)					
	**COICOP code 06.1.1**					
	**M.HERH. Herbal medicines (tablets or syrups) and homeopathic products for consumption outside a health facility or institution.**					
	**M.HERH.PR prescribed**	X	X			
	**M.HERH.OC over-the-counter (self-prescription)**	X	X			
	**M.MVCP. Medicines** (branded, generic)**, vaccines, oral contraceptives, vitamins and minerals for consumption outside a health facility or institution.**					
	**prescribed**					
	**M.MVCP.PR.IA. antibiotics**	X	X			
	**M.MVCP.PR.IO. Other medicines (branded, generic, homeopathic) to treat (presumed or diagnosed) bacterial infections (e.g. malaria, diarrhoeas, dysentery, increased frequency of stools with or without blood and mucus in stools; worms infestations)**	X	X			
	**M.MVCP.PR.CD. medicines to treat (presumed or diagnosed) non-communicable diseases or chronic diseases** (e.g. diabetes, hypertension)	X	X			
	**M.MVCP.PR.FP. oral contraceptives and contraceptives in the form of injections**	X	X			
	**M.MVCP.PR.SY. medicines to treat fevers, pain and other symptoms (e.g. nausea; vomiting, constipation; inflammation)**	X	X			
	**M.MVCP.PR.VM. vitamins, mineral**	X	X			
	**M.MVCP.PR.OM. other prescribed medicines and pharmaceutical preparations not elsewhere specified**	X	X			

	over-the-counter (self-prescription)					
	**M.MVCP.OC.IA. antibiotics**	X	X			
	**M.MVCP.OC.IO Other medicines (branded, generic, homeopathic) to treat bacterial infections (e.g. malaria, diarrhoeas, dysentery, increased frequency of stools with or without blood and mucus in stools; worms infestations)**	X	X			
Examples on NCD should list the most prevalent in country/site	**M.MVCP.OC.CD medicines to treat (presumed or diagnosed) non-communicable diseases or chronic diseases** (e.g. diabetes, hypertension)	X	X			
	**M.MVCP.OC.FP oral contraceptives and contraceptives in the form of injections**	X	X			
	**M.MVCP.OC.SY medicines to treat fevers, pain and other symptoms (e.g. nausea; vomiting, constipation; inflammation)**	X	X			
	**M.MVCP.OC.VM vitamins, mineral**	X	X			
	**M.MVCP.OC.OM other self-prescribed medicines and pharmaceutical preparations not elsewhere specified**	X	X			
	**COICOP code 06.1. 2 medical diagnostic products, prevention and protective devices** for personal use outside a health facility or institution					
	**D.DIAG.** (pregnancy tests; thermometers, glucose-meters, blood pressure meters) **and other medical diagnostic products**	X	X			
	**D. PREP** condoms and other mechanical contraceptive devices, masks , medicinal stockings (e.g. compression stockings), medicinal gloves, insecticide treated mosquito – nets **and other prevention, protective medical devices**	X	X			
	**D.TRTM** inhalers, syringes, humidifiers, nebulizers, hot bags, ice packs, first aid kits, bandages **and other treatment devices for personal use**	X	X			
**Inclusion/Exclusion criteria to be specified when asking about the amount** Only applies to some diagnostic products	Includes repair, rental and maintenance					
	**COICOP code 06.1.3 Assistive products for vision, hearing, mobility and daily living.**					
	**A.PURC.VH. Purchase of** glasses for vision; white canes, glass eyes, contact lenses, hearing aids and **other assistive products for vision and hearing**				X	X
	**A.RRMN.VH. Repair, rental/maintenance of assistive health products for vision and hearing**				X	X
	**A.PURC.MD. Purchase of** crutches & wheelchairs; therapeutic footwear; walkers; pressure relief mattresses **and all other assistive health products for mobility and daily living.**				X	X
	**A.RRMN.MD. Repair, rental/maintenance of assistive health products for mobility and daily living.**				X	X

For peer review only

Supplementary material 3: Supplementary tables

Table 1: Demographic Characteristics of households for matched versus unmatched households and households with versus households without OOPs

	Households with any expenditure		Households without any expenditure		Matched Households		Unmatched Households	
Total number of households	N=2093		N=914		N=1300		N=1698	
	n	%	n	%	n	%	n	%
**Sex**								
Male	1332	64	589	64	841	65	1074	63
**Marital status**								
Married	1326	63	519	57	827	64	1013	60
**Level of Education**								
No education	1116	53	474	52	656	50	931	55
Primary	420	20	196	21	262	20	349	21
Junior high school	292	14	128	14	195	15	223	13
Senior high school	97	5	43	5	71	5	70	4
Vocational/Technical/College/Graduate	167	8	73	8	116	9	125	7
**Religion**								
Christians	1114	53	460	50	730	56	841	50
Islam	162	8	81	9	107	8	136	8
Traditional	708	34	326	34	387	30	638	38
No religion	110	5	47	5	76	6	83	5
**Age group**								
15 - 19	96	5	37	4	60	5	74	4
20-34	138	7	58	6	91	7	102	6
35 - 64	1228	59	543	59	781	60	989	58
65 +	630	30	276	30	368	28	533	31
Mean age (SD)	55	17	55	17	54	17	55	17
**Household size**								
1 person	108	**5**	103	**11**	70	**5**	141	**8**
2-5 persons	1010	**48**	516	**56**	616	**47**	906	**53**
6 and above	975	**47**	295	**32**	614	**47**	652	**38**

For peer review only

Table 2: Summary of mean OOPs for unmatched and matched households by spending categories

	Questionnaire Version 1				Questionnaire Version 2				Questionnaire Version-3			
	11 disaggregated health items				44 disaggregated health items				56 disaggregated health items			
	N	Unmatched Household OOPs Mean (SD)	N	Matched Household OOPs Mean (SD)	N	Unmatched Household OOPs Mean (SD)	N	Matched Household OOPs Mean (SD)	N	Unmatched Household OOPs Mean(SD)	N	Matched Household OOPs Mean (SD)
Outpatient	81	64 (135)	44	66 (128)	181	43 (130)	126	43 (150)	105	44 (78)	47	31 (78)
Inpatient	171	319 (527)	91	226 (279)	177	398 (809)	99	314 (504)	193	287 (716)	100	215 (456)
Medicines	487	41 (140)	302	36 (157)	560	29 (78)	381	25 (56)	609	29 (76)	354	30 (90)
Preventive care	137	59 (95)	80	49 (83)	92	34 (53)	67	31 (53)	46	31 (44)	22	20 (29)
Other medical services	8	203 (201)	2	43 (4)	5	113 (217)	3	15 (10.5)	2	12 (4)	2	12 (4)
Health products	36	71 (133)	19	84 (176)	25	160 (250)	14	178 (289)	18	165 (232)	7	6 (5)

Table 3: Summary of mean OOPs for matched households and provider data by spending categories

	Questionnaire Version 1				Questionnaire Version 2				Questionnaire Version-3			
	11 disaggregated health items				44 disaggregated health items				56 disaggregated health items			
	N	Provider OOPs Mean (SD)	Household OOPs Mean (SD)	Estimated ratio (HH/provider) of the means (95% CI)	N	Provider OOPs Mean(SD)	Household OOPs Mean (SD)	Estimated ratio (HH/provider) of the means (95%CI)	N	Provider OOPs Mean(SD)	Household OOPs Mean (SD)	Estimated ratio (HH/provider) of the means (95%CI)
Outpatient	44	34 (46)	66 (128)	1.92	126	22 (52)	43 (150)	1.98	47	14 (15)	31 (78)	2.23
Inpatient	91	49 (74)	226 (279)	6.5	99	48 (97)	314 (504)	6.54	100	21 (38)	215 (456)	10.34
Medicines	302	26 (67)	36 (157)	1.39	381	21 (57)	25 (56)	1.19	354	12 (21)	30 (90)	2.54
Preventive care	80	32 (47)	49 (83)	1.53	67	38 (75)	31 (53)	0.81	22	12 (18)	20 (29)	1.73
Other medical services	2	11 (11)	43 (4)	4.05	3	16 (7)	15 (10.5)	0.94	2	9 (9)	12 (4)	1.33
Health products	19	44 (61)	84 (176)	1.9	14	21 (28)	178 (289)	8.66	7	36 (31)	6 (5)	0.17

For peer review only

STROBE Statement—checklist of items that should be included in reports of observational studies

	Item No	Recommendation	Page No
**Title and abstract**	1	(*a*) Indicate the study's design with a commonly used term in the title or the abstract	1
		(*b*) Provide in the abstract an informative and balanced summary of what was done and what was found	2
**Introduction**			
Background/rationale	2	Explain the scientific background and rationale for the investigation being reported	4
Objectives	3	State specific objectives, including any prespecified hypotheses	4
**Methods**			
Study design	4	Present key elements of study design early in the paper	6
Setting	5	Describe the setting, locations, and relevant dates, including periods of recruitment, exposure, follow-up, and data collection	5
Participants	6	(*a*) *Cohort study*—Give the eligibility criteria, and the sources and methods of selection of participants. Describe methods of follow-up<br>*Case-control study*—Give the eligibility criteria, and the sources and methods of case ascertainment and control selection. Give the rationale for the choice of cases and controls<br>*Cross-sectional study*—Give the eligibility criteria, and the sources and methods of selection of participants	6
		(*b*) *Cohort study*—For matched studies, give matching criteria and number of exposed and unexposed<br>*Case-control study*—For matched studies, give matching criteria and the number of controls per case	
Variables	7	Clearly define all outcomes, exposures, predictors, potential confounders, and effect modifiers. Give diagnostic criteria, if applicable	7,8
Data sources/ measurement	8*	For each variable of interest, give sources of data and details of methods of assessment (measurement). Describe comparability of assessment methods if there is more than one group	6
Bias	9	Describe any efforts to address potential sources of bias	7,8
Study size	10	Explain how the study size was arrived at	6
Quantitative variables	11	Explain how quantitative variables were handled in the analyses. If applicable, describe which groupings were chosen and why	
Statistical methods	12	(*a*) Describe all statistical methods, including those used to control for confounding	7,8
		(*b*) Describe any methods used to examine subgroups and interactions	
		(*c*) Explain how missing data were addressed	
		(*d*) *Cohort study*—If applicable, explain how loss to follow-up was addressed<br>*Case-control study*—If applicable, explain how matching of cases and controls was addressed<br>*Cross-sectional study*—If applicable, describe analytical methods taking account of sampling strategy	7,8
		(*e*) Describe any sensitivity analyses	

Continued on next page

For peer review only - http://bmjopen.bmj.com/site/about/guidelines.xhtml

For peer review only

**Results**			
Participants	13*	(a) Report numbers of individuals at each stage of study—eg numbers potentially eligible, examined for eligibility, confirmed eligible, included in the study, completing follow-up, and analysed	9
		(b) Give reasons for non-participation at each stage	
		(c) Consider use of a flow diagram	9
Descriptive data	14*	(a) Give characteristics of study participants (eg demographic, clinical, social) and information on exposures and potential confounders	10
		(b) Indicate number of participants with missing data for each variable of interest	
		(c) *Cohort study*—Summarise follow-up time (eg, average and total amount)	
Outcome data	15*	*Cohort study*—Report numbers of outcome events or summary measures over time	
		Case-control study—Report numbers in each exposure category, or summary measures of exposure	
		Cross-sectional study—Report numbers of outcome events or summary measures	11
Main results	16	(*a*) Give unadjusted estimates and, if applicable, confounder-adjusted estimates and their precision (eg, 95% confidence interval). Make clear which confounders were adjusted for and why they were included	13
		(*b*) Report category boundaries when continuous variables were categorized	12,13
		(*c*) If relevant, consider translating estimates of relative risk into absolute risk for a meaningful time period	
Other analyses	17	Report other analyses done—eg analyses of subgroups and interactions, and sensitivity analyses	
**Discussion**			
Key results	18	Summarise key results with reference to study objectives	14
Limitations	19	Discuss limitations of the study, taking into account sources of potential bias or imprecision. Discuss both direction and magnitude of any potential bias	15
Interpretation	20	Give a cautious overall interpretation of results considering objectives, limitations, multiplicity of analyses, results from similar studies, and other relevant evidence	14,15
Generalisability	21	Discuss the generalisability (external validity) of the study results	15
**Other information**			
Funding	22	Give the source of funding and the role of the funders for the present study and, if applicable, for the original study on which the present article is based	16

*Give information separately for cases and controls in case-control studies and, if applicable, for exposed and unexposed groups in cohort and cross-sectional studies.

For peer review only
